# An AIM2 inflammasome biomimetic mineralization inhibitor for vascular dementia therapy

**DOI:** 10.7150/thno.110389

**Published:** 2025-05-25

**Authors:** Yueqi Zhang, Lixian Jiang, Rongrong Wu, Wei Gao, Xiaojie Zhang, Lan Liu, Yaxuan Zhang, Jin Lu, Yuanyi Zheng, Xiaojun Cai, Jianliang Fu

**Affiliations:** 1 Department of Neurology, Shanghai Sixth People's Hospital Affiliated to Shanghai Jiao Tong University School of Medicine, Shanghai 200233, P. R. China; 2 Shanghai Key Laboratory of Neuro-Ultrasound for Diagnosis and Treatment, Shanghai 200233, P. R. China; 3 Department of Ultrasound in Medicine, Shanghai Sixth People's Hospital Affiliated to Shanghai Jiao Tong University School of Medicine, Shanghai 200233, P. R. China; 4 Shanghai Neurological Rare Disease Biobank and Precision Diagnostic Technical Service Platform, Shanghai 200233, P. R. China; 5 Department of Pharmacy, Shanghai Sixth People's Hospital Affiliated to Shanghai Jiao Tong University School of Medicine, Shanghai 200233, P. R. China

**Keywords:** Vascular dementia, AIM2 inflammasome, Prussian blue, pyroptosis, biomimetic mineralization

## Abstract

**Rationale:** Absent in melanoma 2 (AIM2) inflammasome-mediated effector plays critical roles in multiple disease pathologies. While nanotechnology has revolutionized therapeutic development through novel approaches, the potential regulatory effects of nanoparticles on AIM2 inflammasome activity remain unexplored. Here, guided by clinical patient data and computational modeling, we developed an AIM2 inflammasome-targeting biomimetic mineralization inhibitor for vascular dementia (VaD) therapy.

**Methods:** GEO datasets were analyzed to compare AIM2 inflammasome component expression in VaD patient brains versus controls. Molecular dynamics simulations identified high-affinity binding between manganese ferrocyanide and the AIM2 protein. We synthesized hollow manganese Prussian blue nanoparticles (HMPB) via biomineralization and functionalized them with M2 macrophage-derived extracellular vesicles (M2exo@HMPB). Therapeutic efficacy was evaluated in a VaD rat model through intravenous and intracerebroventricular administration, employing behavioral assessments, histopathological analysis, and inflammatory cytokine profiling. AIM2 inflammasome assembly and pyroptosis were investigated through protein immunoblotting, scanning electron microscopy/transmission electron microscopy (SEM/TEM) imaging of microglial cells, and primary microglia cultures under hypoxic-hypoglycemic conditions.

**Results:** Gene expression analysis demonstrated significantly elevated levels of AIM2 inflammasome components in VaD patients compared to normal controls. Molecular dynamics simulations revealed effective binding of manganese ferrocyanide to AIM2. M2exo@HMPB targeted central inflamed sites and were ultimately phagocytosed by microglia. In an *in vitro* sustained hypoxic-hypoglycemic model, M2exo@HMPB inhibited AIM2 inflammasome assembly and pyroptosis in primary microglia, thereby reducing IL-18/IL-1β release and promoting neuronal survival. In VaD rat models, M2exo@HMPB alleviated neuronal loss and white matter lesions while improving learning and executive functions. Additionally, M2exo@HMPB demonstrated favorable *in vivo* biosafety.

**Conclusions**: Integrating clinical bioinformatics with computational drug design, this study establishes a translational paradigm for nanomaterial development. M2exo@HMPB serves not only as an AIM2 inflammasome-targeting biomimetic mineralization inhibitor for VaD therapy but also provides new insights for treating AIM2-mediated cell death pathologies.

## Introduction

Inflammasomes are key factors that regulate cellular stress and immune responses, and their initiation and assembly are closely associated with the occurrence and development of numerous diseases 1. Absent in melanoma 2 (AIM2) is the first inflammasome with a clearly identified ligand. Its ligand, double-stranded DNA (dsDNA), is widely present in the biological context and regionally distributed in eukaryotic cells 2. This makes AIM2 an important cytoplasmic receptor involved in the disrupting intracellular environmental homeostasis and pathogen invasion 3-5. Pathogenic microorganisms, exogenous stimuli, endogenous danger signals, and other factors can activate AIM2 through different mechanisms, triggering its assembly and leading to inflammatory reactions in the body and even irreversible cell death 6. The activation of AIM2 inflammasomes drives the pathogenesis of many aseptic inflammatory diseases, such as cardiovascular diseases, skin diseases, and neuroinflammatory diseases 7. Differential expression of AIM2 has been observed in several autoimmune diseases such as arthritis, psoriasis, atopic dermatitis, venous ulcers, contact dermatitis, colitis, and Sjögren's syndrome, and lupus erythematosus 8. AIM2 inflammasome also plays an adverse role in several types of brain injuries, including ischemic brain injury, cerebral ischemia-reperfusion injury, subarachnoid hemorrhage and traumatic brain injury, generalized anxiety disorder, autism spectrum disorder, and neurodegenerative diseases 9.

Age-related dementia is an irreversible disease that leads to a gradual decline in cognitive abilities and has become one of the major health issues of our time. In China, 1.6% of adults aged 60 or older suffer from Vascular Dementia (VaD), accounting for approximately 26% of all dementia patients 10. Notably, survival time from diagnosis tends to be shorter for individuals with VaD than for those with Alzheimer disease, Lewy body dementia, or frontotemporal lobe degeneration 11. Chronic cerebral hypoperfusion (CCH) is the primary driver of VaD 12 and is strongly associated with stepwise cognitive decline 13. The pathological features of VaD are caused by hypoperfusion, oxidative stress, and inflammation, leading to endothelial damage, blood-brain barrier breakdown, innate immune activation, and disruption of nutritional coupling between blood vessels and brain cells 14. However, treatment with antioxidants, anti-inflammatory drugs, or drugs that increase cerebral perfusion did not lead to consistent results. Some drugs, such as enkephalin (a neurotrophic factor), show moderate cognitive improvement, but the evidence is insufficient to prove clinical use 15. The current clinical strategies for symptomatic treatment of VaD include cholinesterase inhibitors and NMDA antagonists such as memantine. Due to their moderate cognitive improvement and lack of broader benefits, they are considered unsuitable for VaD treatment 16. Therefore, discovering new therapeutic targets for VaD is urgent and of great significance. AIM2, a sensor of double-stranded DNA (dsDNA), could be driven by endogenous dsDNA released from damaged host cells 4. Microglia-induced AIM2 production mediated cognitive impairment pathogenesis, and AIM2 inflammasome-dependent pyroptosis contribute to acute and chronic neuronal death after stroke 17,18. Genetic deletion of AIM2 attenuated cognitive deficits in AD and VaD models 19. Therefore, AIM2 is a potential therapeutic target in VaD.

Regulating AIM2 inflammasome activation is crucial for controlling inflammatory responses and cell apoptosis. In recent years, the impact of AIM2 inflammasome-mediated pyroptosis on disease has received increasing attention. Most of the drugs found to inhibit the function of AIM2 inflammasomes come from natural products, such as andrographolide 20, fructose arginine 21, quercetin 22, and so on. However, there are still shortcomings, such as low bioavailability and a short effective blood drug concentration time at the target site. Nanobiological interactions could yield unexpected and very interesting results, such as black phosphorus nanomaterials, which inhibit the intrinsic biological activity of PLK1 kinase on mitotic centrosome instability 23; FDA-approved ferumoxytol for iron deficiency treatment showing anti-leukemia effect 24; and graphene oxide nanosheets, which selectively inhibit the intrinsic biological activity of DNMT3A-mutated leukemia cells 25. Compared to the small molecule drugs (such as the reported AIM2 inflammasome inhibitors including andrographolide 20, fructose arginine 21, quercetin 22), these bioactive nanoparticles exhibit special advantages, such as (1) being engulfed or adhered by cells, resulting in a long effective blood drug concentration time at the target site; and (2) good biological stability, as they are not easily degraded by proteins in the body, maintaining long-term activity. However, it is still unknown whether nanomaterials have tunable AIM2 inflammasome regulatory activity.

**Figure 1:**
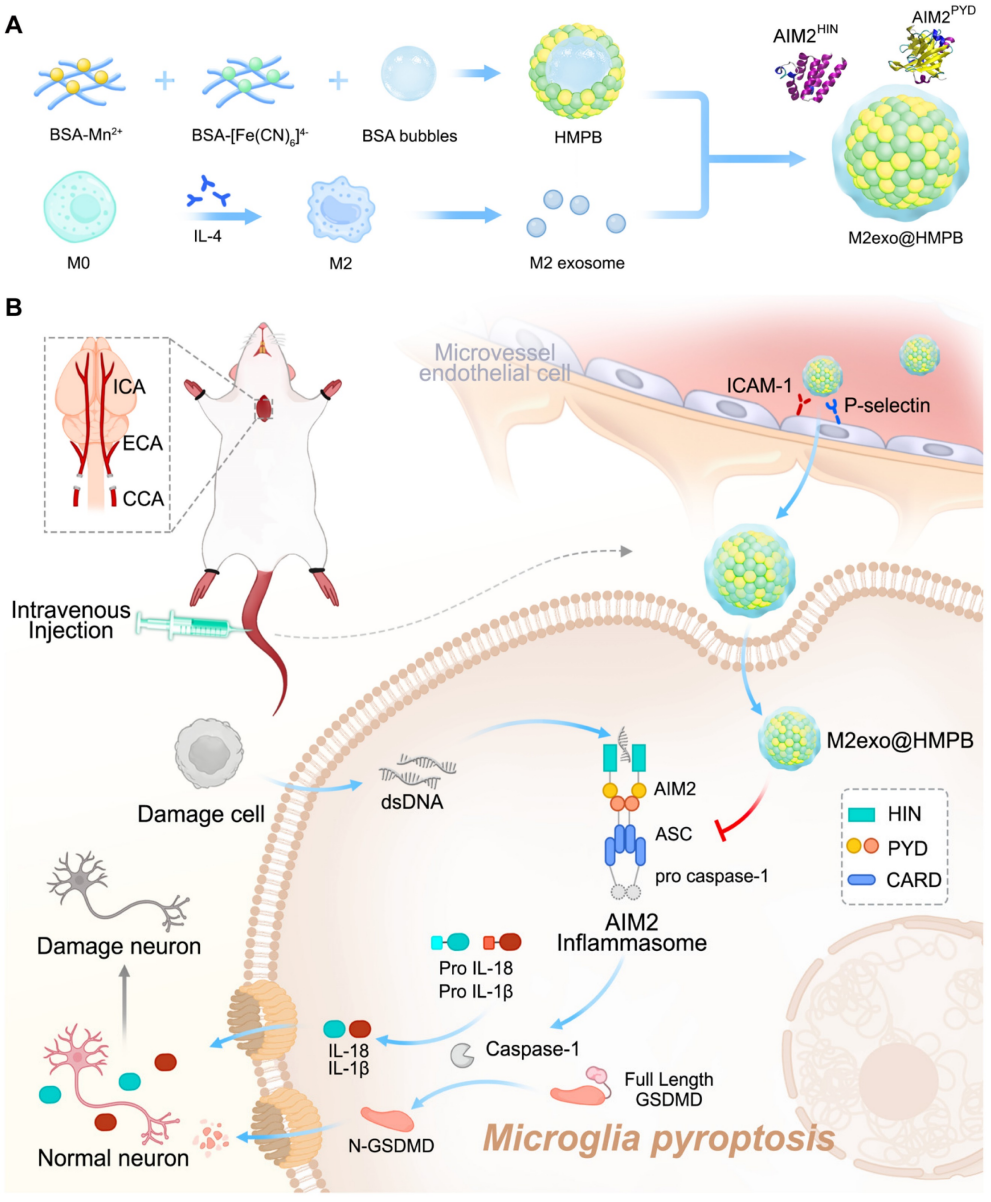
**Regulatory effects of M2exo@HMPB on AIM2 inflammasome signaling. (A)** Schematic of M2exo@HMPB synthesis. **(B)** Enhanced blood-brain barrier (BBB) penetration through CD11b/ICAM-1 and CD44/P-selectin ligand-receptor interactions facilitates microglial uptake of M2exo@HMPB. This nanocomposite suppresses AIM2 inflammasome activation, mitigates pyroptosis, and reduces proinflammatory cytokine release, thereby exerting neuroprotective effects in a VaD rat model.

Herein, we reported that an AIM2 inflammasome biomimetic mineralization inhibitor was developed for VaD therapy, guided by clinical patient sample information and theoretical computational simulations (**Figure 1**). We first performed gene expression analysis of the components of the AIM2 inflammasome in the brain tissue of patients with VaD, which demonstrated higher levels of AIM2 in patients with VaD than in normal controls. AIM2 may be as a therapeutic target for VaD. Molecular dynamics (MD) simulations were adopted to find that manganese ferrocyanide can effectively bind to AIM2, thereby inhibiting its activity and potentially becoming a novel AIM2 inflammasome inhibitor. Guided by the results of molecular dynamics simulations, we employed the biomineralization method to obtain hollow manganese Prussian blue nanoparticles (HMPB) with excellent biocompatibility (**Figure 1A**). In order to achieve the adhesion and recruitment of HMPB in AIM2 inflammasome overexpression diseases, as well as to evade capture by the reticuloendothelial system, we selected extracellular vesicles of M2 macrophages to modify HMPB (M2exo@HMPB). M2exo@HMPBs were injected intravenously or intracerebroventricularly to inhibit AIM2 inflammasome-mediated pyroptosis in microglia, thereby rescuing neuronal loss and improving the learning and executive function of VaD rats. M2exo@HMPB only reduced the overexpression of AIM2 inflammasomes to normal levels, rather than completely inhibiting them. M2exo@HMPB has no effect on the expression of normal AIM2 inflammasomes (**Figure 1B**). This study demonstrates that the AIM2 inflammasome plays a critical role in VaD by operating through caspase-1/gasdermin D-dependent pyroptotic pathways. This study provides not only an unprecedented treatment option for VaD, but also a foundation and new insights for the treatment of AIM2-mediated cell death diseases, bringing new opportunities of nanotechnology and nanomedicine.

## Results and Discussion

### MD simulation guided the design of the AIM2 inhibitor

**Figure 2:**
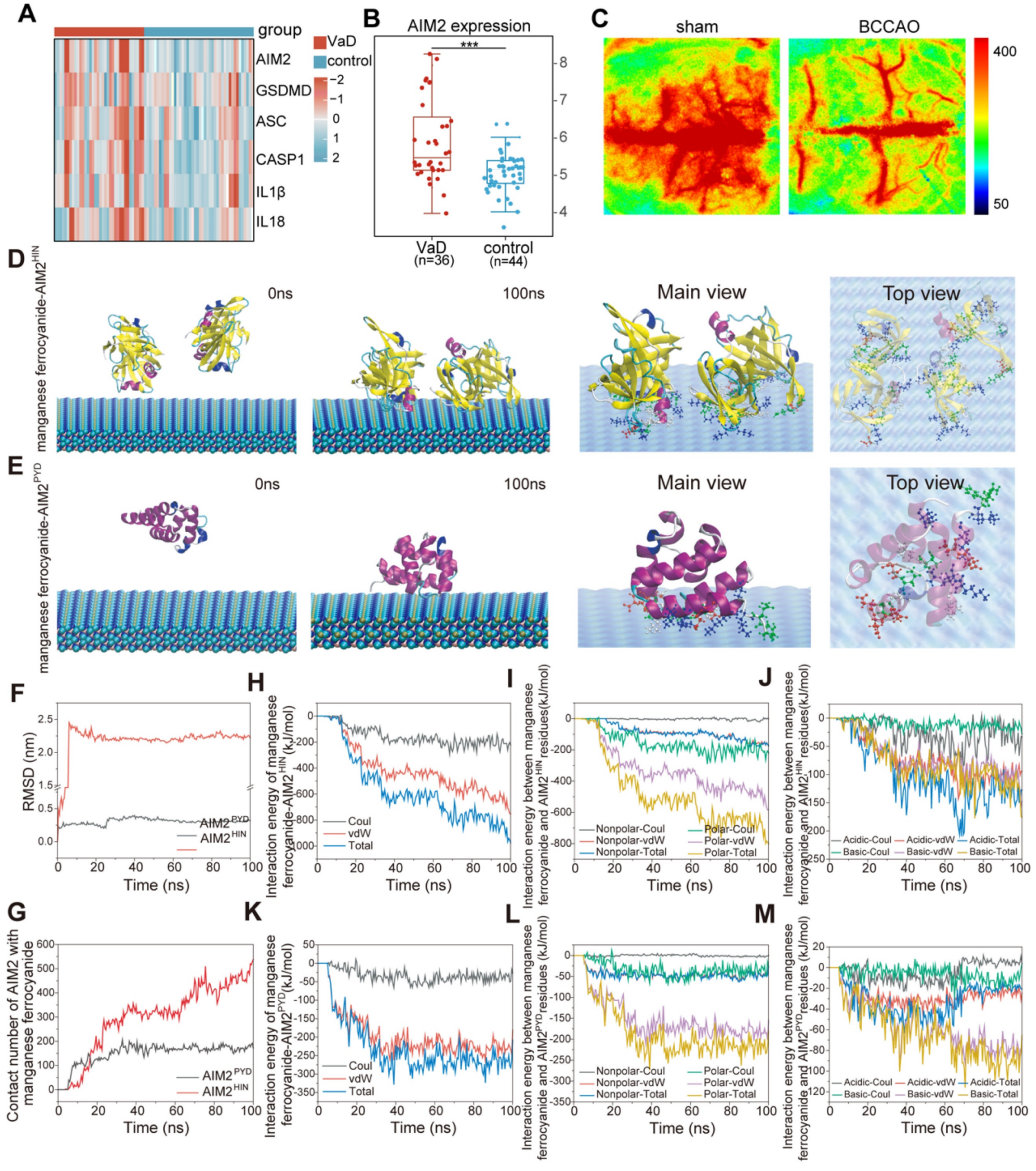
**Clinical patient sample information and MD simulation-guided design of AIM2 inhibitors. (A)** Heatmap upregulated AIM2 inflammasome components (AIM2, ASC, Caspase-1, IL-1β, and IL-18) in brain tissues of VaD and healthy patients from the GEO database (GSE122063). **(B)** AIM2 mRNA levels in the brain of VaD and healthy patients from the GEO database. **(C)** BCCAO rat model mimicking CCH in VaD. Representative laser speckle contrast imaging. n = 3 per group. **(D-E)** MD simulations of manganese ferrocyanide binding to AIM2 domains: Initial/final conformations and interfacial residue analysis (nonpolar: white; polar: green; acidic: red; basic: blue) for AIM2^HIN^ (D) and AIM2^PYD^ (E). **(F)** RMSD of proteins values changes during simulations. **(G)** Atoms contact number of AIM2^HIN^ and AIM2^PYD^ with manganese ferrocyanide, which means atoms within 0.6 nm of manganese ferrocyanide. **(H-M)** Interaction energy profiles: Total binding energy (H, K), residue-specific contributions (nonpolar/polar: I, L; acidic/basic: J, M).

To determine whether the AIM2 inflammasome is activated during VaD pathogenesis, we analyzed AIM2 inflammasome component expression in VaD patient brain tissues using GEO datasets (GSE122063). Gene expression analysis of AIM2 inflammasome components revealed elevated levels in VaD patients compared to normal controls, demonstrating its potential as a therapeutic target for VaD (**Figure 2A-2B**). A rat model of bilateral common carotid artery occlusion (BCCAO) was established to mimic CCH, as observed in patients with VaD. BCCAO surgery successfully induced VaD in rats, reducing cerebral blood flow (CBF) to approximately 50% of baseline levels (**Figure 2C**).

All-atoms MD simulations were performed to investigate AIM2-manganese ferrocyanide interactions. Both major functional domains of AIM2—the C-terminal domain (AIM2^HIN^, containing 200 amino acid repeats) and the N-terminal domain (AIM2^PYD^)—were stably adsorbed by manganese ferrocyanide, as shown in initial and final conformations (**Figure 2D-E**). Root mean square deviation (RMSD) analysis (**Figure 2F**) indicated minimal conformational changes in AIM2^PYD^, whereas AIM2^HIN^ exhibited significant deviation, which can also be observed from the conformation snapshot in **Figure 2D-E**. Contact analysis within 0.6 nm of manganese ferrocyanide atoms demonstrated that AIM2^HIN^ rapidly adhered to manganese ferrocyanide (0-23 ns), remained stable (23-62 ns), and further interacted post-62 ns (**Figure 2G**). Similarly, AIM2^PYD^ rapidly adhered within 0-10 ns and maintained a stable conformation thereafter.

Total interaction energy between manganese ferrocyanide and AIM2^HIN^ reached -976.6 kJ/mol, predominantly contributed by van der Waals (-753.33 kJ/mol) and electrostatic (-223.3 kJ/mol) forces (**Figures 2H and 2K**). The three types of interaction energies between manganese ferrocyanide and AIM2^PYD^ were -208.20 kJ/mol, -181.31 kJ/mol, and -26.89 kJ/mol. Furthermore, the specific residues of two AIM2 domains in contact with manganese ferrocyanide were deeply analyzed and classified. As shown in **Figure 2D**, the interact residues between the AIM2^HIN^ portion and manganese ferrocyanide mainly include non-polar residues (PRO, LEU, VAL, ILE, ALA, and PHE), polar residues (THR, GLN, ASN, GLY, SER, and TYR), basic amino acids (GLU and ASP), and acidic amino acids (LYS, ARG and HIS). From the data of the interaction energies of the four parts (**Figure 2I-J**), it can be seen that the interaction between polar residues and manganese ferrocyanide contributes significantly to the overall adsorption, and the interaction energies of acidic and basic residues reach -127.33 kJ/mol and -175.80 kJ/mol. The residues involved in the interaction between the AIM2^PYD^ domain and the manganese ferrocyanide, as shown in **Figure 2E**, exhibit similarities to those found in the AIM2^HIN^ domain (**Movie S1 and S2**). The analysis of interaction energy (**Figure 2L-M**) showed that the interaction energy of polar amino acids (TYR, THR, ASN, GLN) can ultimately reach -181.45 kJ/mol, which also play a more important role in adsorption process than nonpolar residues of -26.46 kJ/mol (LEU and ILE). And the interaction energy between basic amino acids (LYS and ARG) and manganese ferrocyanide was stronger than that between acidic amino acids (GLU and ASP), which refer to -65.48 kJ/mol and -20.49 kJ/mol. In summary, the MD results demonstrate that manganese ferrocyanide has the ability to adsorb AIM2 protein, especially with stronger interactions between the AIM2^HIN^ domain. These findings support its potential as an effective AIM2 inhibitor.

### Construction and characterization of the AIM2 inhibitor

**Figure 3:**
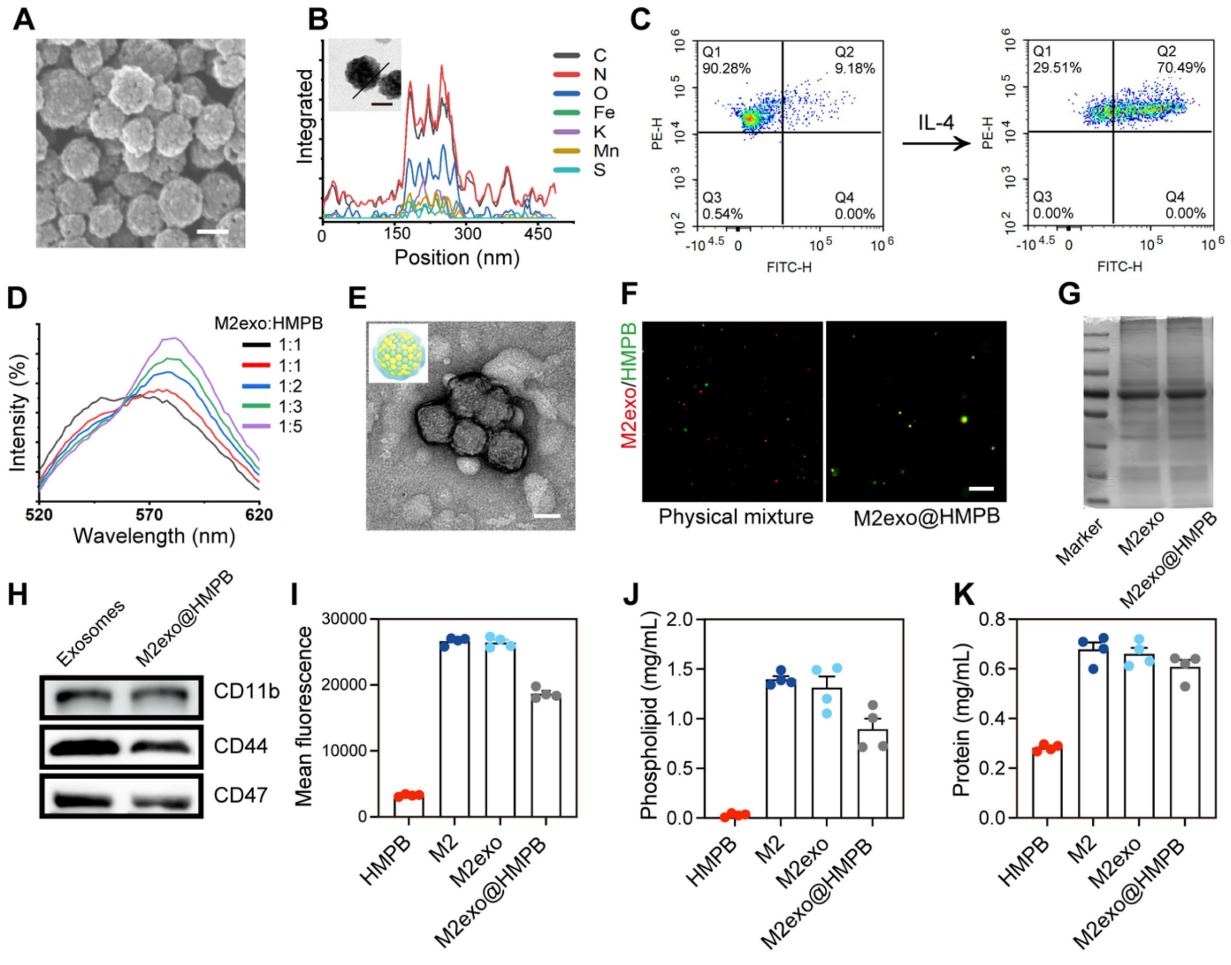
** Design and characterization of M2exo@HMPB. (A)** SEM image of HMPB. Scale bar: 100 nm. **(B)** Line element (C, O, N, K, Fe, Mn, and S) mapping and HAADF image of HMPB. Scale bar: 100 nm. **(C)** Flow cytometry quantification of CD68^+^/CD206^+^ cells post-IL-4 treatment. **(D)** FRET analysis of M2exo@HMPB integration. **(E)** Representative TEM image of exosomes. Scale bar: 100 nm. **(F)** Fluorescent colocalization of exosome (red) and HMPB (green) in a physical mixture or fused M2exo@HMPB. Scale bar: 20 μm. **(G)** SDS-PAGE protein profiling. **(H)** Quantification of CD47, CD11b, and CD44 in the exosomes and M2exo@HMPB. **(I)** Orientation of glycoproteins via WGA staining. **(J)** Quantitative content analysis of phospholipids, and **(K)** protein. n = 4 independent experiments. Data: mean ± SEM.

To address disease requirements, we synthesized HMPBs via biomineralization with excellent biocompatibility, guided by MD simulations (**Figure 1A**). High-angle annular dark-field (HAADF) imaging and elemental mapping demonstrated the coexistence of Fe, Mn, K, C, N, O, and S in the prepared HMPB** (Figure 3A-B and Figure S2)**. Transmission electron microscopy (TEM, **Figure S1A**) and scanning electron microscopy (SEM, **Figure 3A**) revealed uniform monodisperse HMPB. The constructed HMPB had X-ray diffraction characteristic peaks consistent with those of manganese Prussian blue, without other scattered peaks (**Figure S1B**). Fourier transform infrared spectroscopy exhibited characteristic Fe-CN-Mn peaks and protein absorption bands (**Figure S1C**). XPS results showed binding energy peaks of Fe and Mn (**Figure S1D**). M2 macrophages are recognized as an anti-inflammatory phenotype. They preferentially migrate to sites of injury or chronic inflammation, where they facilitate tissue repair and mediate immune regulation. Previous studies reported M2 macrophages are more motile and migrate over longer distances towards chemoattractants relevant for neuroinflammation compared to M0 and M1 macrophages 26. M2 exosomes exhibit elevated expression of adhesion molecules such as LFA-1 and VLA-4 27. These surface markers enable specific binding to ICAM-1/VCAM-1 overexpressed on inflamed epithelial cells. To achieve adhesion and recruitment of AIM2 inflammasome nanomodulators in diseases with AIM2 inflammasome overexpression, as well as to evade capture by the reticuloendothelial system, we selected exosomes of M2 macrophages to modify AIM2 inflammasome inhibitors (**Figure 1A**). To obtain exosomes derived from M2 macrophages, IL-4 was used to induce the differentiation of M0 to M2 macrophages.

Flow Cytometry results demonstrated that the proportion of CD68/CD206-positive cells increased from 9.18% to 70.49%, indicating that the majority of macrophages had been polarized to the M2 phenotype (**Figure 3C**). TEM and nanoparticle tracking analysis verified the morphology and particle size of M2exo (**Figure S3A-B**). Western blot confirmed exosomal markers CD9, CD81, and CD63, with Calnexin as a negative control (**Figure S3C**). Using ultrasonic fusion and extrusion methods, HMPB encapsulated in M2 macrophage exosomes was obtained (denoted as M2exo@HMPB). Dynamic light scattering (DLS) analysis indicated a particle size close to 160 nm for HMPB, with a slight increase post-membrane modification (**Figure S4A-B**). Zeta potentials were measured as -14.7 mV for HMPB and -18.7 mV for M2exo@HMPB (**Figure S4C**). Förster resonance energy transfer (FRET) pairs (C6-NBD/DHPE-RhB) confirmed the fusion through fluorescence intensity changes (**Figure 3D**). TEM verified the morphology of M2exo@HMPB (**Figure 3E**)**.** Fluorescent co-localization of M2exo (red) and HMPB (green) in M2exo@HMPB contrasted with distinct signals in physical mixtures (**Figure 3F**), collectively confirming successful encapsulation. Ensuring that the phospholipid proteins of M2 exosomes remain on the surface of M2exo@HMPB is crucial for exerting their biological functions. Quantitative analysis demonstrated successful transfer of phospholipids and proteins from M2 exosomes to M2exo@HMPB (**Figure 3G, 3J-K**). The protein composition of M2exo@HMPB was determined by sodium dodecyl sulfate-polyacrylamide gel electrophoresis (SDS-PAGE). M2exo@HMPB retained almost all the major proteins present on M2 exosome membranes, including key proteins such as CD47, CD11, and CD44, as demonstrated by protein imprinting (**Figure 3H**). The correct orientation of membrane proteins is also crucial for their function. The orientation of M2exo@HMPB glycoproteins was determined using fluorescently labeled wheat germ agglutinin (WGA) as a model. WGA-labeled macrophages, M2 exosomes, and M2exo@HMPB confirmed the correct orientation of glycoproteins (**Figure 3I**). The above results fully demonstrate the successful construction of M2exo@HMPB.

### Lesion localization and targeting efficacy of the AIM2 inhibitor

**Figure 4:**
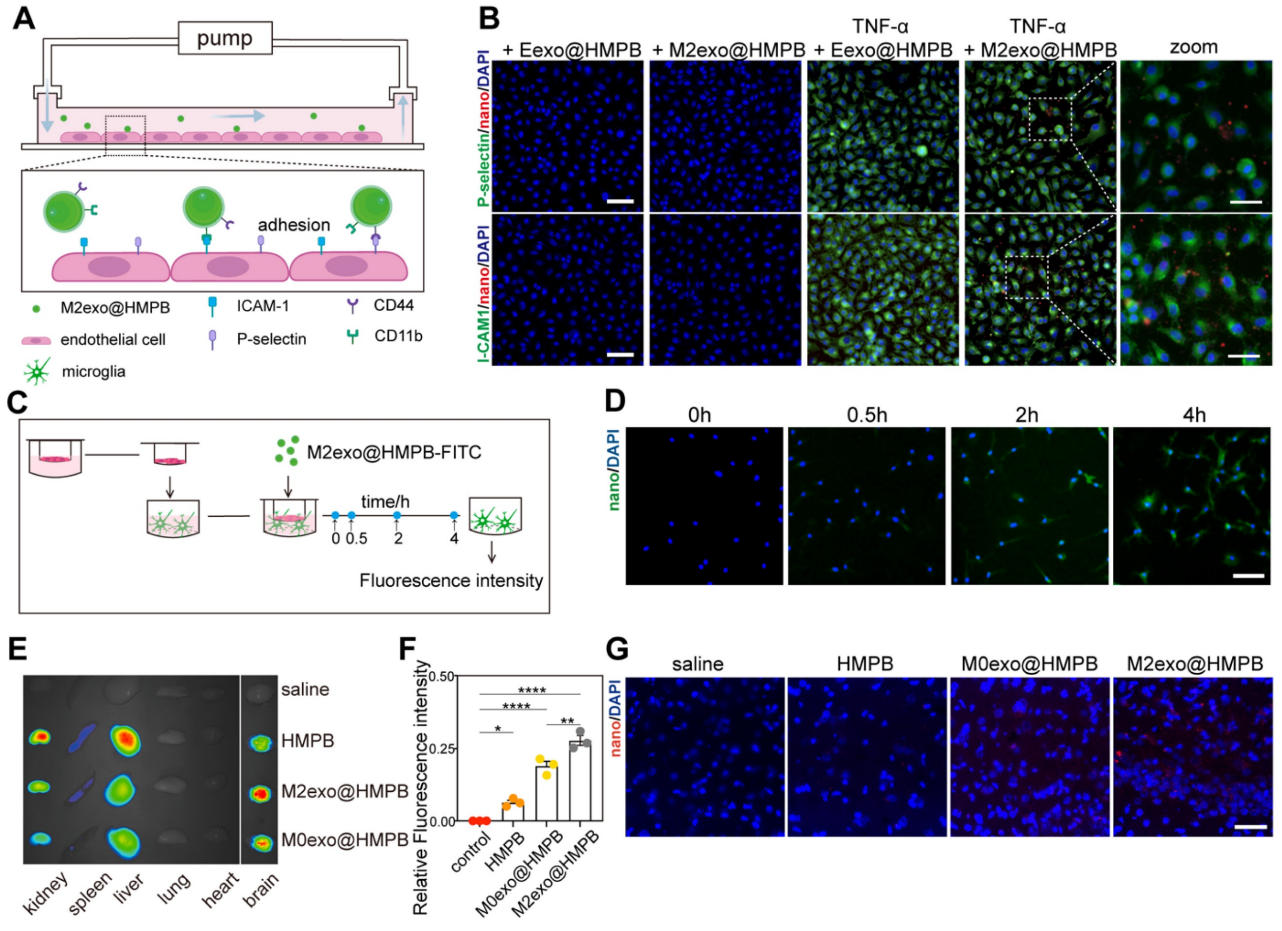
** BBB penetration and biodistribution of M2exo@HMPB. (A-B**) *In vitro* adhesion assay: Microvessel model schematic and Dil-labeled nanoparticles (red)/epithelial markers (green). Scale bar: 100 μm (left), 50 μm (right). n = 3 independent experiments. **(C)** Trans-BBB migration assay: Schematic illustration of the *in vitro* BBB model and basal chamber FITC signal. Scale bar: 100 μm. n = 3 independent experiments. (**E-F**) *In vivo* biodistribution: Fluorescence imags and relative fluorescence intensity of the brain and major organs of CCH rats at 48 h after injection with saline or Cy5.5-labeled nanoparticles. Data: mean ± SEM. n = 3 independent experiments. **(G)** Representative fluorescence images of sections of CCH rat brains at 48 h after injection with saline or Cy5.5-labeled nanoparticles. Scale bar: 100 μm. n = 3 per group.

To assess lesion localization and targeting efficacy, we analyzed M2exo@HMPB adhesion using a versatile pump system mimicking blood-brain barrier (BBB) microvasculature under VaD conditions (**Figure 4A**). Inflammation-induced specific binding constitutes the primary mechanism for macrophages targeting brain injury sites. In this process, inflammation-activated blood vessels express high levels of adhesion molecules such as P-selectin and ICAM-1, which are bound by macrophages through membrane adhesion factors such as CD44 and CD11b. Therefore, M2exo@HMPB likely recognizes damaged vasculature via high-affinity interations with CD44 and CD11b. TNF-α-treated HUVECs exhibited marked upregulation of ICAM-1 (**Figure 4B**, green) and P-selectin (**Figure 4B**, green) compared to untreated HUVECs. Correspondingly, M2exo@HMPB (red) exhibited stronger binding to TNF-α-treated HUVECs. This enhanced binding may result from upregulated adhesion molecule expression and augmented molecular interactions between M2exo@HMPB and HUVECs. Red blood cell membrane vesicles loaded with HMPB (Eexo@HMPB) served as a control. The binding of M2exo@HMPB to TNF-α-treated HUVECs was significantly higher than that of Eexo@HMPB (**Figure 4B**). Ligand-receptor recognition between CD11b/ICAM-1 and CD44/P-selectin may be one of the mechanisms underlying the specific binding of M2exo@HMPB to damaged cells. These results confirm that the characteristic proteins derived from macrophages can help M2exo@HMPB target damaged brain microvessels. Then, we evaluated the ability of M2exo@HMPB to traverse the BBB using a Transwell System (**Figure 4C**). Permeability was quantified as the fluorescence intensity ratio (basal/upper chamber) of culture medium at four time points. The fluorescence intensity ratio progressively increased over the observed period, indicating cumulative diffusion of M2exo@HMPB across the BBB *in vitro* (**Figure S5A**). Fluorescence images revealed that M2exo@HMPB (green) traversed the simulated BBB, entered the lower chamber, and was phagocytosed by microglia (**Figure 4D**).

We successfully established a VaD rat model by performing BCCAO surgery, in which the cerebral blood flow (CBF) decreased significantly to approximately 50% of the baseline (**Figure S6**). To evaluate the delivery, distribution, and targeted accumulation of M2exo@HMPB *in vivo*, Cy5.5 labeled-HMPB or -M2exo@HMPB nanoparticles were administered via the tail vein to VaD or sham rats. After 24 hours, major organs were collected for imaging, and brain tissue sections were prepared for analysis. Brain fluorescence imaging showed significantly higher M2exo@HMPB accumulation compared to uncoated or M0 membrane-coated HMPB (**Figure 4F**). Meanwhile, main organ fluorescence imaging revealed that M2exo@HMPB accumulated much less in the liver and spleen, indicating that the M2exo membrane facilitated the reticuloendothelial system evasion of nanoparticles. This likely prolonged circulation time, enhancing central accumulation (**Figure 4E**). Significant fluorescence of M2exo@HMPB was observed in the brain tissue sections of CCH rats after intravenous injection (**Figure 4G**), indicating the penetration of M2exo@HMPB into the inflamed parenchyma. To evaluate the BBB transport ratio of M2exo@HMPB *in vivo*, Mn content of brain tissue was quantified by ICP-MS before and 24 h after administration. The *in vivo* BBB transport ratio of M2exo@HMPB was calculated as 10%. Furthermore, to analyze the differential kinetic profile and retention efficiency of M2exo@HMPB in the central nervous system depending on the route of delivery, a comparative analysis of Mn levels in brain tissue was conducted 24 hours post-administration via intravenous versus intracerebroventricular administration (**Figure S5B**). **Figure S7** illustrates the long-term metabolic behavior of M2exo@HMPB *in vivo*, demonstrating its gradual and sustained biodegradation within the brain parenchyma. These results demonstrate the ability of M2exo@HMPB to target the central inflamed sites conferred by their M2-derived exosome membrane coating. This binding is probably attributable to specific interactions between chemokine receptors on the alternatively activated M2 macrophage membrane and chemoattractants released by inflamed cells 26.

### Inhibition of AIM2 inflammasome activity* in vitro*

**Figure 5:**
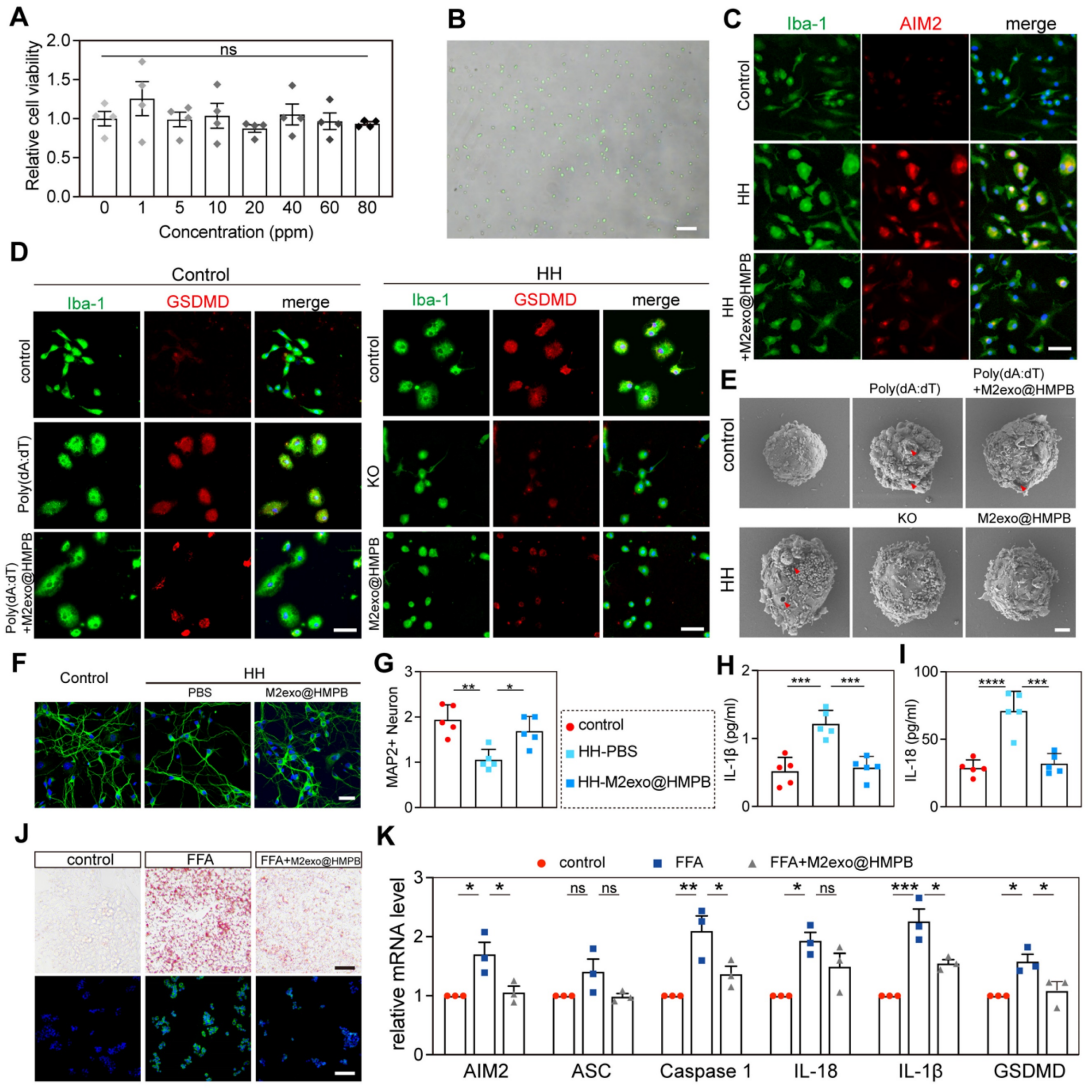
** M2exo@HMPB modulates AIM2 inflammasome activity *in vitro*. (A)** Relative cell viability of primary rat microglia after incubation with different concentrations of M2exo@HMPB. n = 4 independent experiments. **(B)** Transfection efficiency of AIM2-KO lentivirus in primary microglia. **(C-D)** AIM2 (red)/Iba-1 (green) and GSDMD (red)/Iba-1 co-staining. Scale bar: 50μm. **(E)** Representative SEM analysis showing the cell morphology in different treatment groups. Red arrowhead: membrane pores and blebs. Scale bar: 2 μm. **(F-G)** MAP2/DAPI-stained neurons and quantification. **(H-I)** ELISA-detected IL-1β/IL-18 levels. n = 4 independent experiments.** (J-K)** Lipid droplet staining (Oil Red O/Bodipy; scale: 100 μm) and AIM2-related mRNA expression in HepG2 (n=4; mean±SEM). *P<0.05; **<0.01; ***<0.001; ns=non-significant.

We next investigated the inhibitory effect of M2exo@HMPB on AIM2 inflammasome activity *in vitro*. M2exo@HMPB (10-80 ppm) exerted no significant cytotoxicity toward primary rat microglia (**Figure 5A**). A sustained hypoxic-hypoglycemic (HH) model with AIM2 inflammation activation was established. Significant morphological changes in microglial cells following sustained HH, including cell swelling and reduced protrusions, markedly increased AIM2 immunofluorescence intensity (**Figure 5C**).

After 48 h of HH stimulation, the viability of primary rat microglia was markedly reduced (**Figure S8**). Live-dead staining revealed that M2exo@HMPB significantly increased the green fluorescence in microglial cells (**Figure S9**). M2exo@HMPB significantly inhibited AIM2 expression (**Figure 5C**). Cationic liposomes (LyoVec^TM^) encapsulating poly(deoxyadenylic-deoxythymidylic) acid sodium salt (poly[dA:dT]) were used to specifically induce AIM2 inflammasome activation. Microglia were exposed to poly(dA:dT) to specifically activate AIM2 inflammasome and induce pyroptosis. Lentivirus-mediated AIM2 knockout was employd to inhibit AIM2 inflammasome formation, allowing comparison of protective effects between genetic ablation and M2exo@HMPB treatment against HH-induced pyroptosis. Increased enhanced green fluorescent protein (EGFP) expression, as measured by fluorescence microscopy, confirmed the transfection efficiency (**Figure 5B**). The expression of the key pyroptotic effector protein, GSDMD, was analyzed by immunofluorescence staining. The fluorescence intensity of GSDMD significantly increased after exposure to poly(dA:dT) (**Figure 5D**). However, M2exo@HMPB notably reduced the expression of GSDMD and largely restored the cell morphology. Similarly, under HH conditions, microglial cells swelled and GSDMD expression markedly increased. However, both AIM2 knockout and M2exo@HMPB treatment significantly reduced GSDMD levels, alleviating the pathological morphological changes induced by HH in microglia. SEM was used to evaluate microglial membrane integrity, as membrane pores induced by GSDMD are a key feature of pyroptosis. These images (**Figure 5E**) show that after prolonged exposure to HH, microglia swelled and significant pores appeared on the membrane, similar to the alteration induced by poly(dA:dT). Additionally, M2exo@HMPB markedly reduced membrane pores under HH conditions. These findings indicate that M2exo@HMPB inhibited the AIM2 inflammasome induced by hypoxic-hypoglycemia in microglia.

Because neuronal loss is the primary direct cause of chronic neurological diseases, proinflammatory cytokines can induce neuronal cell death in culture and *in vivo* 28. Thus, we assessed the neuroprotective effect of M2exo@HMPB by evaluating its ability to suppress the toxic effects of IL-1β and IL-18 released from microglia pyroptosis. In the microglia-neuron co-culture system (**Figure S10**), the neurons co-cultured with microglia pretreated with HH exhibited significantly lower MAP2 fluorescence intensity than did those in the control group. These neurons also exhibited shorter or discontinuous axons. However, these abnormal changes were not evident in neurons co-cultured with microglia pre-treated with M2exo@HMPB thereafter (**Figure 5F-G**). Additionally, the levels of IL-1β and IL-18 in the culture supernatant were consistently reduced (**Figure 5H-I**). M2exo@HMPB significantly reduced the levels of inflammatory cytokines released into the extracellular environment of the coculture system. Taken together, these findings suggest that M2exo@HMPB treatment suppresses microglia inflammasome activation and pyroptosis, decreases the release of inflammatory factors, and promotes neuronal survival *in vitro*. Furthermore, we compared M2exo@HMPB with Probenecid, a conventional AIM2 inflammasome inhibitor reported in prior studies 7, 29, in the *in vitro* CCH model. The results demonstrated that both M2exo@HMPB and Probenecid significantly inhibited AIM2 inflammasome expression in microglial cells under HH conditions (**Figure S11A**). Additionally, both treatments effectively inhibited the release of inflammatory cytokines IL-18 and IL-1β, with no statistically significant difference in efficacy observed between the two interventions (**Figure S11B-C**). In a non-alcoholic fatty liver disease (NAFLD) *in vitro* model, free fatty acid (FFA)-stimulated hepatocytes exhibit AIM2 inflammasome activation concomitant with cellular steatosis 30. Thus, we mimicked hepatic steatosis induced by FFA overload in HepG2 cells and examined the effects of M2exo@HMPB on AIM2 inflammasome activation and steatosis. As expected, Oil Red O and BODIPY 493/503 staining revealed significant lipid accumulation in HepG2 cells following FFA treatment. However, M2exo@HMPB significantly diminished lipid deposition in the hepatocytes (**Figure 5J**). As shown in **Figure 5K**, M2exo@HMPB significantly inhibited the expression of AIM2 inflammasome components compared to the negative control in FFA-induced HepG2 cells, as determined by qPCR. These results demonstrate an M2exo@HMPB regulatory effect on the AIM2 inflammasome, suggesting a potential therapeutic role for M2exo@HMPB in diseases involving the AIM2 inflammasome.

### Improvement of cognitive deficits in VaD rats

**Figure 6:**
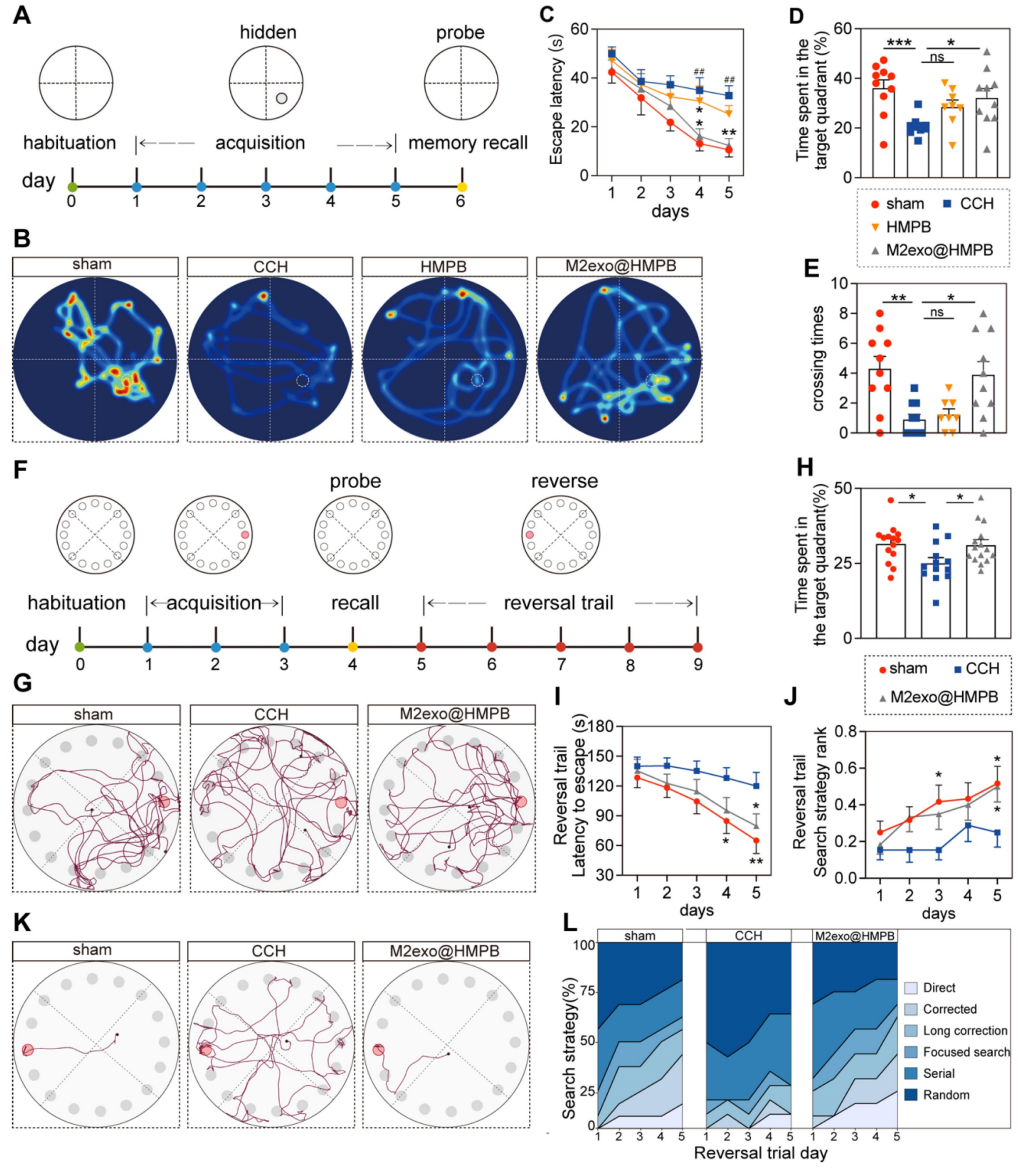
** M2exo@HMPB improved cognitive deficits in CCH rats. (A)** Experimental design of Morris water maze. **(B)** Swimming paths of rats in different groups during the probe trial. **(C)** Escape latency of rats to reach the hidden platform during the acquisition test, n = 8-10 per group. **(D)** Time spent in the target quadrant of rats in the probe trial, n = 8-10 per group. **(E)** Platform crossings during the probe trial, n = 8-10 per group. **(F)** Experimental design of the Barnes maze. **(G)** Exploration traces of rats in different groups during the probe trial. **(H)** Percentage of time spent in the target quadrant by the rats in the probe trial, n = 13-15 per group. **(I)** Escape latency of the rats to find the escape box during the reversal trial, n = 13-15 per group. **(J)** Search strategies used by the rats during the reversal trial, n = 13-15 per group. **(K)** Representative search traces of the rats in different groups on the last day of the reversal trial. **(L)** Distribution of search strategies used by rats in different groups on the reversal days. Data: mean ± SEM. *P < 0.05, **P < 0.01, ***P < 0.001 vs. CCH; ^#^P < 0.05, ^##^P < 0.01, ^###^P < 0.001 vs. sham; ns: not significant.

To evaluate the protective effect of M2exo@HMPB against CCH-induced cognitive deficits, spatial learning and memory were assessed using the Morris Water Maze (MWM) after 10 weeks of treatment (**Figure 6A**). BCCAO-induced hypoperfusion significantly prolonged escape latency during the learning phase (**Figure 6C**). CCH rats exhibited impaired learning capacity, with significantly prolonged escape latency on the 4th and 5th days compared to sham controls. M2exo@HMPB treatment significantly decreased the time spent locating the hidden platform, whereas HMPB showed limited efficacy (**Figure 6C**). In the probe trial, M2exo@HMPB-treated CCH rats exhibited a remarkably higher target quadrant preference and platform crossings compared to saline controls (**Figure 6B**, and **Figure 6D-E**). The above results indicate that M2exo@HMPB improved the cognitive impairment induced by CCH. Additionally, it exerted a better treatment effect than did HMPB on the learning and memory performance of CCH rats. Therefore, our subsequent behavioral experiments focused mainly on the M2exo@HMPB-administered group.

To assess executive function and cognitive flexibility, rats underwent Barnes maze testing (**Figure 6F**). The treatment with M2exo@HMPB significantly increased the percentage of time that CCH rats spent in the target quadrant, where the escape box was located (**Figure 6H**). Movement tracking showed CCH rats adopted random exploration, while sham and M2exo@HMPB-treated groups focused on the target quadrant with escape box-directed search behavior (**Figure 6G**). Reversal learning trials were conducted to evaluate cognitive flexibility. A significant reduction in escape latency was observed during Barnes maze reversal-learning phase (**Figure 6I**). The administration of M2exo@HMPB significantly improved escape latency on the fifth day of the reversal experiment (P < 0.05) (**Figure 6I**). M2exo@HMPB significantly improved the search strategies of CCH rats, as evidenced by a significant increase in the proportion of advanced search strategies (**Figure 6J and Figure 6L**). Collectively, M2exo@HMPB enhances spatial learning and memory capabilities, executive function and cognitive flexibility in CCH rats.

### The therapeutic efficacy of M2exo@HMPB in VaD rats

**Figure 7:**
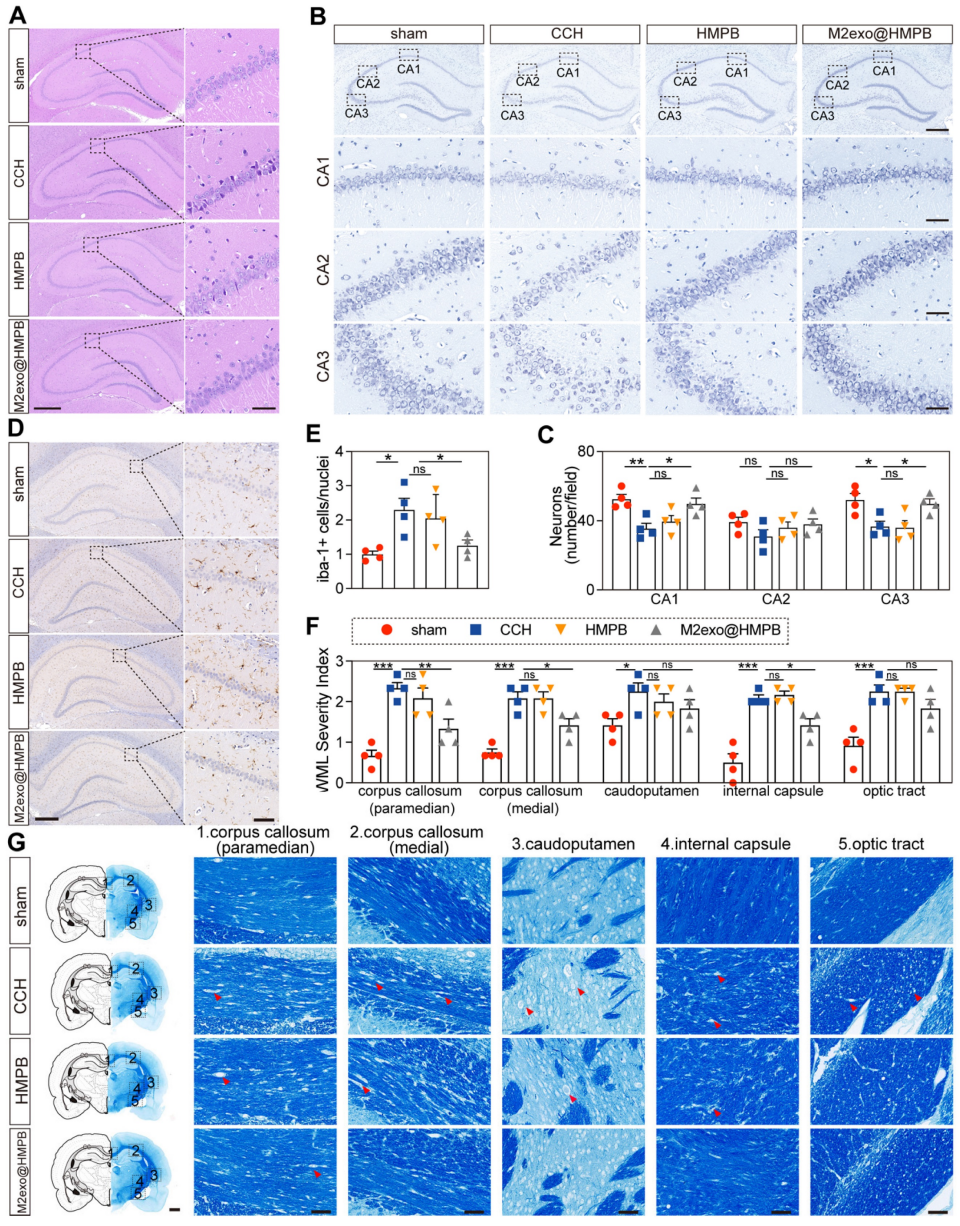
** Effect of M2exo@HMPB on the levels of glial activation, white-matter integrity, and hippocampal neuronal density in the hippocampus in CCH rats. (A)** Representative H&E staining of the rat hippocampus. Scale bar: 500 μm (left panel) or 50 μm (right panel), n = 3 per group. **(B-C)** Nissl staining and neuronal quantification in CA1/CA2/CA3 regions. Scale bar: 500 µm (upper panel) or 50 μm (lower panels), n = 4 per group. Representative immunostaining of Iba-1 **(D)** and quantification of Iba-1-positive microglia **(E)** in the rat hippocampal region. Scale bars: 500 μm (left panel) or 50 μm (right panel), n = 4 per group. Representative Luxol fast blue staining **(F)** and quantification of white-matter integrity **(G)** in the corpus callosum (paramedian), corpus callosum (medial), caudoputamen, internal capsule, and optic tract in rats. Scale bars: 1mm (left panel) or 50μm (right panels), n = 4 per group. The data (C, E, F) are presented as the mean ± SEM. *P < 0.05; **P < 0.01; ***P < 0.001 vs. CCH. ns means not significant.

The hippocampus, a crucial center of cognition and memory, is involved in the encoding, storage, and retrieval of information, which play a critical role in learning new knowledge and forming long-term memories 31. To assess whether M2exo@HMPB had a protective effect on the hippocampal neurons of CCH rats, Hematoxylin and eosin (H&E) and Nissl staining of hippocampal sections were performed for histological analysis. H&E staining revealed well-organized neurons with intact nuclei in sham controls, whereas chronic cerebral hypoperfusion (CCH) induced hyperchromasia and karyopyknosis, indicative of neuronal damage (**Figure 7A**). Furthermore, Nissl staining indicated neuronal loss and disorganized architecture in CCH rats, consistent with neurodegeneration (**Figure 7B**). This damage was improved by M2exo@HMPB in the treatment groups, which was more pronounced in the hippocampal CA1 and CA3 areas (**Figure 7C**). Hippocampal CA1 and CA3 regions contribute to the formation of the classic trisynaptic pathway, which is an important neural connection for learning and memory 32. However, HMPB treatment did not significantly alleviate neuronal necrosis in CCH rats. These staining results are consistent with the behavioral experimental outcomes.

Microglial activation, a hallmark of CCH and neuroinflammation 33, exacerbates neuronal dysfunction and cognitive decline. The microglia-specific marker Iba-1 was examined. We found that microglia in the hippocampus region exhibited small soma and ramified processes in the sham group. In contrast, the number of Iba-1+ microglia significantly increased in the CCH group, displaying an activated morphology characterized by enlarged cell bodies with rarefied and shortened processes (**Figure 7D**). The number of microglia in the M2exo@HMPB-treated group was significantly lower than that in the CCH group (**Figure 7E**). However, HMPB treatment had no significant effect on the morphology or number of microglia in the hippocampus of CCH rats. These results suggest that M2exo@HMPB can significantly alleviate the abnormal activation of microglia in CCH rats.

White matter lesions impair neural signal transmission efficiency and correlate with cognitive decline 34. White matter alteration is a prominent feature of VaD. The white matter integrity analysis revealed varying degrees of white matter rarefaction in the CCH group (**Figure 7G**). However, M2exo@HMPB significantly improved certain areas of CCH rats. The severity scores of white matter lesions showed that this improvement was primarily evident in the corpus callosum and internal capsule (P < 0.05). HMPB treatment also showed an improvement in the caudoputamen, but this trend was not statistically significant (P ≥ 0.05). These results indicate that M2exo@HMPB treatment can significantly alleviate white matter damage in CCH rats and improve the integrity of the white matter (**Figure 7F**). Thereby, improving cognitive impairment and alleviating the hallmark neuropathological alterations associated with VaD, including neuronal loss, white matter lesions, and abnormal microglial activation.

In the intravenous injection treatment study, HMPB exhibited limited therapeutic effects compared to M2exo@HMPB. This could be attributed to clearance by the circulatory system and the barrier function of the BBB, which prevent HMPB from exerting its expected therapeutic effects. In clinical practice, patients are more likely to receive intravenous administration. For chronic diseases, lateral ventricular injection of nanomodulators has several advantages: (1) BBB bypass for localized delivery; (2) prolonged retention via cellular uptake; and (3) minimized systemic toxicity. In addition, intraventricular injection has been widely used in clinical practice. Similar to the intravenous administration, the spatial learning and memory performance of M2exo@HMPB treated rats were significantly better than those of the untreated CCH group. Intravenous administration of HMPB did not result in significant outcomes, whereas administration through the lateral ventricle led to partial neuropathological amelioration (**Figure S12 and S13**). Major organ histology (H&E) and serum biochemistry confirmed the biosafety of M2exo@HMPB (**Figure S14**).

### Therapeutic mechanism of M2exo@HMPB in VaD rats

**Figure 8:**
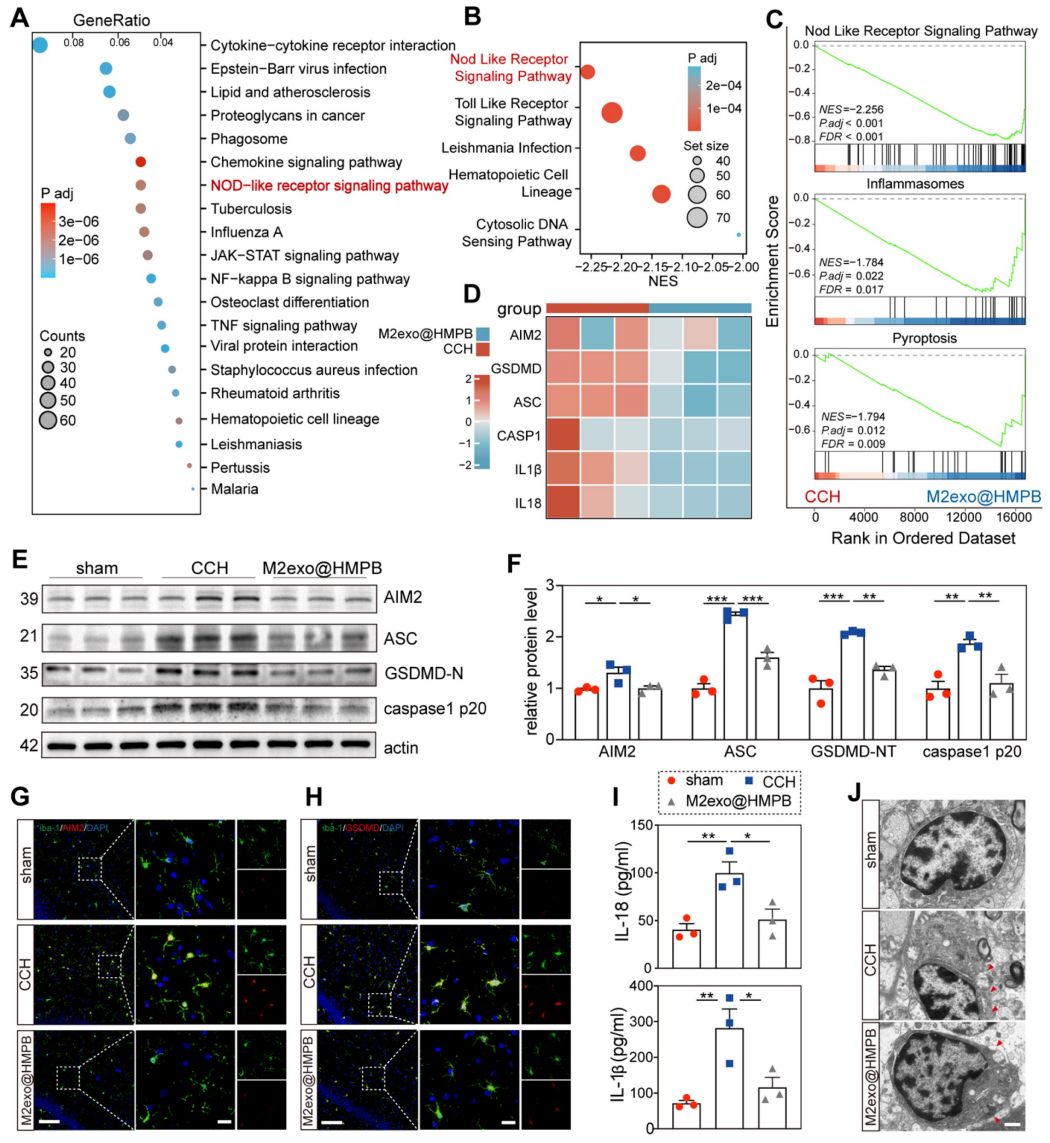
** M2exo@HMPB inhibited AIM2 inflammasome in CCH rats. (A)** The intersection of DEGs between M2exo@HMPB and CCH was analyzed using KEGG enrichment, and the top 20 enriched pathways are shown, n = 3 per group. **(B)** Top five enriched KEGG pathways in the GSEA. **(C)** Enrichment plots from GSEA of genes. NES, normalized enrichment score. FDR, false discovery rate. |NES| > 1, FDR q-value < 0.25 were considered different between the two groups. n = 3 per group. **(D)** Heatmap indicating differential mRNA expression of the components of AIM2 inflammasome (AIM2, ASC, Caspase-1, IL-1β, and IL-18) in the hippocampus tissues of CCH and M2exo@HMPB-treated rats. **(E)** Representative immunoblotting bands and **(F)** quantification of AIM2, ASC, GSDMD-N, and caspase-1 p20 in the rat hippocampus, n = 3 per group. **(G-H)** Immunofluorescence staining of AIM2/Iba-1 and GSDMD/Iba-1 co-localization in the rat hippocampus. Scale bar: 100 μm (left panel) or 20 μm (right panel), n = 3 per group. **(I)** IL-1β and IL-18 levels in the rat hippocampus, n = 3 per group. **(J)** Representative TEM images of microglia in the rat hippocampus. The red arrowhead indicates membrane pores. Scale bar: 1 μm, n = 3 per group. Data: mean ± SEM. *P < 0.05; **P < 0.01; ***P < 0.001 vs. CCH. ns: not significant.

Principal component analysis (PCA) of mRNA expression data revealed distinct transcriptomic profiles between the CCH and M2exo@HMPB-treated groups, as evidenced by separated score scatter plots (**Figure S15A**). Volcano plots identified 399 upregulated and 1179 downregulated differentially expressed genes (DEGs) in CCH rats treated with M2exo@HMPB (**Figure S15B**). Kyoto Encyclopedia of Genes and Genomes (KEGG) analysis of DEGs between saline- and M2exo@HMPB-treated CCH rats highlighted the NOD-like receptor signaling pathway as a top enriched pathways (**Figure 8A**). Additionally, Gene Set Enrichment Analysis (GSEA) identified the NOD-like receptor signaling pathway as one of the most enriched pathways in M2exo@HMPB (**Figure 8B**). GSEA showed suppression of the NOD-like receptor signaling pathway, inflammasome, and pyroptosis in M2exo@HMPB-treated rats (**Figure 8C**), implicating these pathways in VaD therapeutic efficacy. The key genes related to AIM2 inflammasome assembly and pyroptosis were further screened. As expected, several significant DEGs involved in this process were down-regulated in the M2exo@HMPB group (**Figure 8D**). Western blotting was performed to further evaluate the expression of pyroptosis-related proteins in the hippocampus. As depicted in **Figure 8E-F**, CCH rats exhibited elevated levels of AIM2, GSDMD-N, caspase-1 p20 (the active form of caspase-1), and ASC in the hippocampus. However, these increases were reversed by M2exo@HMPB, which was consistent with the results of transcriptomic analysis. ELISA further demonstrated significant reduction of IL-1β and IL-18 in M2exo@HMPB-treated CCH rats (**Figure 8I**). A database of purified cell types from the brains of wild-type mice revealed that AIM2 is expressed in various types of CNS cells, with particularly high expression levels in microglia 35. These data suggest that microglia are important targets for M2exo@HMPB treatment. Double immunofluorescence staining of AIM2/Iba-1 and GSDMD/Iba-1 revealed increased AIM2^+^ and GSDMD^+^ microglia in CCH rats, which was mitigated by M2exo@HMPB (**Figure 8G-H**). Pyroptosis is a lytic mode of cell death that is dependent on GSDMD-induced membrane pore formation 5. Therefore, TEM was used to observe alterations in the microglial membrane following CCH and M2exo@HMPB treatment. As shown in **Figure 8J**, the number of membrane pores in the microglia was markedly increased in CCH rats; however, M2exo@HMPB administration mitigated this abnormality. Taken together, these data support the notion that CCH induces pyroptotic cell death in microglia, and M2exo@HMPB inhibits this process by suppressing AIM2 inflammasome.

## Conclusion

This study utilized clinical patient sample information and theoretical computational simulations (molecular dynamics simulations) to guide the development of AIM2 inhibitors, demonstrating a referenceable research paradigm for nanomaterials. M2exo@HMPBs serve as an AIM2 inflammasome biomimetic mineralization inhibitor for VaD therapy. In cell models with AIM2 inflammasome activation, including HH and NAFLD, the inhibitory effect of M2exo@HMPBs on AIM2 inflammasomes was confirmed using agonists and gene knockouts. Furthermore, in a VaD model with AIM2 inflammasome overexpression, M2exo@HMPB inhibited the activation of AIM2 inflammasomes and cell pyroptosis in microglia, thereby alleviating chronic cerebral hypoperfusion-induced neuronal cell death and cognitive impairment. Unlike existing nanoparticles that rely on direct central administration to bypass BBB and possess limited biodegradability, M2exo@HMPB, through a fabric strategy involving both top-down and bottom-up approaches, provides a lesion-targeting capability driven by physiopathological functions of natural cell membrane and effectively inhibits inflammation. Given the central role of AIM2 inflammasome in inflammatory pathologies, M2exo@HMPB—a biomimetic mineralization inhibitor—holds therapeutic potential beyond VaD, including neurodegenerative and autoimmune diseases. This study validates AIM2 inflammasome as a therapeutic target for VaD and proposes a translatable strategy for AIM2-associated disease management.

## Methods

### Preparation and characterization of HMPB

Potassium ferrocyanide (17.8 mg) and bovine serum albumin (100 mg) were dissolved in 10 mL of water to form solution A. Manganese chloride (10 mg) and bovine serum albumin (100 mg) were dissolved in 10 mL of water to obtain solution B. Solution A was dropwise added to solution B under magnetic stirring at room temperature for 2 h to obtain a white solution. This solution was then stored at 4 °C for 12 h, centrifuged at 13,000 rpm for 15 min, and washed with deionized water to obtain the HMPB.

The structure and morphology of HMPB were analyzed by TEM and SEM. Elemental maps were generated by STEM (JEOL, JEM-3200FS, Japan). XRD patterns were recorded using a diffractometer with Cu-Kα radiation (Rigaku-Ultima-IV, Japan). XPS spectra were obtained with a spectrometer under Al Kα excitation (K-Alpha, Thermo Scientific, USA). Protein components and functional groups in HMPB were analyzed by FT-IR spectrometer (IR Affinity-1S, Shimadzu Kyoto, Japan).

### Exosome isolation

Macrophage-derived exosomes were isolated as described previously. NR8383 rat macrophage cells (Cell Bank, Chinese Academy of Sciences, Shanghai, China) were stimulated with IL-4. Supernatants were stepwise centrifuged at 300 ×g and 2000 ×g for 10 min to remove cells and debris, then at 10,000 g for 30 min to further eliminate debris. The final supernatant was ultracentrifuged at 100,000 g for 70 min twice to pellet exosomes, which were resuspended in PBS and stored at -80 °C. Exosome morphology was imaged by TEM (FEI Talos F200X G2, USA). The zeta potential and particle size were analyzed using nanoparticle tracking analysis (Particle Metrix ZetaView Nano, Germany). Exosomal markers were identified by Western blotting.

### Preparation and characterization of M2exo@HMPB

M2exo@HMPB was prepared using ultrasonic fusion technology. Initially, 2 mL 1 mg/mL HMPB was mixed with 0.2 mg M2exo and sonicated for 3 min in an ice bath by an ultrasonic device (75W, SB-5200DT, SCIENTZ, China). Finally, the mixture was extruded through polycarbonate membranes (800 nm and 400 nm pore sizes) to obtain M2exo@HMPB particles approximately 150 nm in size.

### HMPB and M2exo fusion analysis

The fusion was analyzed by Förster Resonance Energy Transfer (FRET). Acridine orange and rhodamine B were respectively loaded into HMPB and exosomes. Samples were mixed at ratios of 5:1, 3:1, 2:1, and 1:1, then sonicated for 3 min, samples were analyzed using a microplate reader (Tecan Spark 20M, Swiss) with 460 nm excitation (emission: 500-650 nm). For verification, FITC-labeled HMPB and DiI-labeled exosomes were sonicated for 3 min, extruded through an 800 nm nucleopore hydrophilic membrane. 3 μL of M2exo@HMPB suspension was fixed on glycerol gelatin-coated slides. The simple physical mixture without the fusion process served as control. Fluorescence images were captured by confocal laser scanning microscope (A1s, Nikon, Japan).

### Characterisation of the M2exo@HMPB

The particle size distribution and zeta potential of M2exo@HMPB were measured using dynamic light scattering (Zetasizer Lab, Malvern, UK) at room temperature in triplicate. The stability of M2exo@HMPB in PBS was assessed through daily particle size measurements over a period of seven days. The structure and morphology of M2exo@HMPB were analyzed by TEM (FEI Talos F200X G2, Thermo scientific, USA) after staining with 1% uranyl acetate.

The protein composition M2exo@HMPB was analyzed via SDS-PAGE (10% gel) and western blotting. Samples (30 µg) were denatured at 100 °C for 5 min in loading buffer, electrophoresed, and imaged (AI800, Cytova, USA). Characteristic markers of the exosomes (CD9, CD63, CD81 and negative marker Calnexin) and key proteins associated with macrophage adhesion (CD11b and CD44) and immune recognition (CD47) were identified by Western blotting. Proteins were transferred to PVDF membranes and incubated overnight at 4 °C with the following primary antibodies: CD9 (1:1000, Cat#A19027, Abclonal, China); CD63 (1:1000, Cat#52090, Cell Signaling Technology, USA); Calnexin (1:1000, Cat#2679, Cell Signaling Technology, USA); CD81 (1:1000, Cat#A24484, Abclonal, China); CD11b (1:1000, Cat#17800, Cell Signaling Technology, USA); CD44 (1:200, Cat#GB112054, ServiceBio, China); and CD47 (1:1000, Cat#A21904, Abclonal, China). Membranes were incubated with secondary antibody (1:10000, Jackson ImmunoResearch Laboratories, USA) for 2 h at room temperature and visualized by an ECL kit.

### Adhesion assessment

Human umbilical vein endothelial cells (HUVECs) were seeded into a flow chamber (µ-Slide I0.4 Luer, ibidi, Germany) and maintained in DMEM (containing 10% FBS). When the cells reached 80-90% confluence, they were stimulated with recombinant TNF-α (50 ng/mL) for 6 h to upregulate the expression of P-selectin and ICAM-1. Despite lacking inflammation-targeting proteins (CD44 and CD11b), M2exo@HMPB exhibited macrophage-like membrane characteristics, we assessed the targeting efficacy of M2exo@HMPB by comparing it with red blood cell membrane vesicles loaded with HMPB (Eexo@HMPB), which served as a control. Subsequently, DiI-labeled Eexo@HMPB and M2exo@HMPB (10 ppm) were perfused at a shear rate of 500 s^-1^ for 15 min, followed by a 5-min wash. The colocalization density of HMPB or M2exo@HMPB nanoparticles with P-selectin and ICAM-1 was quantified using fluorescence microscopy (BZ-X810, Keyence, Japan).

### BBB penetration assessment

A total of 1 × 10^5^ HUVECs were seeded into Transwell inserts (12 mm diameter, 1.0 µm pore size) and incubated in 12-well plates at 37 °C for 14 days. A vacuum pump was used to remove air bubbles. BBB tight junction integrity was confirmed via trans-endothelial electrical resistance (TEER) measurement of the co-culture model using a Millicell-ERS device, ensuring 100% confluence after 14 days. The inserts were then transferred to a 12-well plate containing primary microglia and equilibrated at 37 °C for 1 day. Subsequently, 20 µL of FITC labeled M2exo@HMPB at a concentration of 1 mg/mL was added to the inserts. Microglial uptake of FITC-M2exo@HMPB in the lower chamber was monitored at different time points using fluorescence microscopy.

*In vitro* BBB transport ratio was calculated as the fluorescence intensity ratio (basal/upper chamber) of culture medium by multimode microplate reader (Tecan Spark, Switzerland). The BBB transport ratio of M2exo@HMPB and route comparative analysis *in vivo* were determined by the brain Manganese (Mn) content to the total administered dose, accounting for the total blood volume in rats: Transport ratio = Brain Mn Content × Total Blood Volume in Rats/Total Mn in Injected M2exo@HMPB Solution. SD rats with a mean body weight of 250 g were utilized in this study. The total blood volume was estimated as 15 ml per rat. CCH rats received intravenous tail vein injection of M2exo@HMPB 300 μL or intracerebroventricular injections of M2exo@HMPB 6 μL. Brains were harvested before injection and 24 h after injection and subjected to vascular perfusion with PBS. Mn content was evaluated by ICP-MS (7900, Agilent, USA).

The metabolic profile of M2exo@HMPB *in vivo* was evaluated by measuring the Mn content rats brains at 1, 24, 72, and 168 h following intracerebroventricular injection of 6 μL M2exo@HMPB. Mn content was evaluated by ICP-MS (7900, Agilent, USA).

### Molecular dynamics simulation

To investigate the interaction between HMPB and AIM2 protein, two simulation systems were constructed: HMPB-AIM2HIN and HMPB-AIM2PYD, modeling interactions between HMPB and the HIN/PYD domains of AIM2 (denoted by HMPB-AIM2^HIN^ and HMPB-AIM2^PYD^ system). The two domains of AIM2^HIN^ and AIM2^PYD^ were obtained from the PDB database (PDB ID: 3RN2 and 4O7Q). The Amber99SB-ILDN force field was selected for protein parameters. HMPB was modeled in Materials Studio, with force field parameters generated via Sobtop and partial charges assigned using the PACMAN online tool. The charge parameters were generated using PACMAN online tool. Simulation box dimensions were 9.90 × 8.36 × 10.28 nm^3^ and 9.20 × 6.27 ×7.71 nm^3^, and the initial distance between the HMPB and AIM2 domains was 1.5 nm. TIP3P water molecules and Na^+^/Cl^-^ ions were added to balance the charge of the simulation system. Periodic boundary conditions were set in three directions X, Y, and Z. In particular, HMPB was fixed during the whole simulation process. Hydrogen bonded interactions were constrained using the LINCS algorithm.

After the energy minimization process using a steepest descent algorithm with a maximum of 5000 steps, NVT equilibration was performed and further adopted for the whole simulation. The simulation time step was set at 2 fs, and a final 100 ns production run was conducted for each system. During the whole simulations, an appropriate thermostat of V-rescale to maintain constant temperature at 310 K. The nonbonded interaction and short-range electrostatic interactions were calculated with a cut-off value of 1.0 nm, and the Particle Mesh Ewald (PME) algorithm was used for the electrostatic interactions. The entire simulation was completed using GROMACS 2019.6. VMD software was used to visualize the simulation results.

### Experimental animals

Sprague‒Dawley rats were purchased from the Beijing Charles River Laboratory Animal Technology Co., Ltd. All animals were housed in specific pathogen-free facilities. All experimental procedures in this study were performed in accordance with the principles outlined in the National Institutes of Health (NIH) Guide for the Care and Use of Laboratory Animals and were approved by the Charles River Institutional Animal Care and Use Committee (P2022046).

Animals were randomly assigned to either the sham-operated group or the CCH surgery groups at the beginning of each experiment. Animals in the CCH group were randomly assigned to receive either drug treatments or vehicle control. Animals were coded with numbers, and experimenters were blinded to the treatment assignment.

### CCH rat model and treatment

CCH rat model was established via BCCAO. Briefly, the rats were anesthetized with sodium pentobarbital (50 mg/kg, i.p.). Each common carotid artery was doubly ligated with a 4-0 silk suture (Covidien, Mansfield, MA, USA) and transected between the ligations. Sham rats underwent the same bilateral common carotid artery exposure without occlusion of the arteries. Rats received weekly intravenous injections of HMPB or M2exo@HMPB (15 mg/kg) for 10 weeks, or a single ICV injection of 8 μL. The control groups were treated with the same volume of saline as vehicle.

### ICV stereotactic injection

Rats were anesthetized using pentobarbital and secured in a manual-guided stereotaxic apparatus (RWD Life Science, China). A 1 mm hole was drilled 0.80 mm posterior and 1.5 mm lateral to the bregma. The needle was inserted through the hole to a depth of 3.5 mm (targeting the right lateral ventricle). A total volume of 8 μL of HMPB or M2exo@HMPB or saline was infused using a microliter syringe (1701, Hamilton, USA) connected to a motorized nanoinjector (Single Syringe Nanoliter Infusion/Withdraw Pump, Legato 130, KD Scientific Inc., USA). The injection rate was set as 0.5 μL/min. The needle was left in the injection site at least for 10 min after administration.

### Morris water maze test

The Morris water maze (MWM) test was conducted to assess spatial learning and memory abilities. Rats exhibiting clearly water aversion or inability to swim were excluded. A circular pool (50 cm in height, 150 cm in diameter) filled with water (23±1 °C, depth 45 cm) was used. The pool was divided into four quadrants with different visual cues, and a submerged platform (diameter 10 cm, 2 cm below water surface) was placed in the third quadrant center. The orientation phase consisted of four trials a day for five consecutive days. Each trial initiated from a different quadrant, allowing rats 60 s to locate the platform. Rats that located the platform were allowed to remain for 10 s; those that failed were gently guided to it and kept there for 10 s. Escape latency was recorded. A probe trial was performed on the sixth day, during which rats were allowed to navigate freely for 60s without the platform. Data including the number of platform crossings, time spent in the target quadrant (%), and swimming speed were analyzed using EthoVision® XT 15.

### Barnes Maze test

All rats were naïve to prior behavioral testing. Barnes maze (Maze Engineers, Boston, MA, USA) testing was conducted in a behavioral suite equipped with spatial cues and an aversive light source, except training trials. EthoVision XT (Noldus, Wageningen, Netherlands; version 11.5) was utilized to collect and analyze video data. Following training to the location of the escape, the rats learned the task across 3 days, with 2 trials per day. Spatial memory was assessed 3 days later, in one probe trial. Reversal learning (5 days, 2 trials/day) to assess executive function began the following day, during which the location of the escape hole was changed without additional training. Rats were assessed for their latency to find the escape hole, and search strategies were manually categorized by an investigator blinded to group assignments as follows: direct (1), corrected (0.75), long correction (0.5), focused search (0.5), serial (0.25), and random (0).

### Laser speckle contrast imaging

Before and after undergoing BCCAO surgery, rats were secured in a stereotaxic apparatus to minimize movement and prevent motion artifacts during imaging. The skull was carefully thinned and polished in the area stretching from the front fontanelle's coronal line to the lambdoid suture's coronal line using a drill with a diameter of 1.5 mm until the cerebral surface vasculature became faintly visible. To enhance the clarity of the images, the prepared skull area was moistened with glycerol. Subsequently, the rats were positioned for analysis under a laser probe. The PeriCam PSI HD system (Perimed, Datavägen 9 A, Sweden) was utilized to assess variations in cortical blood flow before and after BCCAO surgery. The entire polished brain area was taken as the region of interest and each rat was monitored for 5 min.

### Immunofluorescence staining

For *in vivo* fluorescence staining, deparaffinized sections underwent antigen retrieval (0.05% citraconic acid), endogenous peroxidase blocking (3% H₂O₂ in PBS, 10 min), and blocking (5% donkey serum containing 0.1% Triton X-100 in PBS, 1 h). Sections were incubated overnight at 4 °C with the following primary antibodies: anti-AIM2 (1:200, Cat#20590-1-AP, Proteintech, China), anti-GSDMD (1:1000, Cat#GB114198, Servicebio, China), Iba-1 (1:500, Cat#66827-1-Ig, Proteintech, China), followed by corresponding fluorescent secondary antibodies. Nuclei were stained with DAPI for 5 min at room temperature. For *in vitro* fluorescence staining, cells fixed with 4% paraformaldehyde for 15 min were permeabilized and blocked (5% donkey serum containing 0.1% Triton X-100 in PBS, 1 h), incubated overnight at 4 °C with antibodies as follows: AIM2 (1:200, Cat#20590-1-AP, Proteintech, China), GSDMD (1:200, Cat#GB114198, Servicebio, China), Iba-1(1:200, Cat#5076, Abcam, UK), then incubated with secondary antibodies for 1 h. Images were acquired using a fluorescence microscope (IX53, Olympus, Tokyo, Japan) or a confocal microscope (Nikon, Tokyo, Japan).

### Enzyme-linked immunosorbent assay (ELISA)

The concentrations of interleukin (IL)-1β (Cat#70-EK201B/3, Multisciences, Hangzhou, China) and IL-18 (Cat#70-EK282/4, Multisciences, Hangzhou, China) in the hippocampal tissue homogenates and culture medium supernatants were quantified using ELISA kits following the manufacturer**'**s instructions.

### Real-time PCR

Total RNA from HepG2 cells was extracted using the RNAeasy™ Animal RNA Isolation Kit (QIAGEN, German) with a spin column according to the manufacturer**'**s protocol. Isolated RNA was reverse-transcribed into cDNA using the PrimeScript™ RT Master Mix (Perfect Real Time) (Takara Bio, USA) following the standard protocol. The qPCR assay was conducted using TB Green™ Premix Ex Taq™ II (Tli RNaseH Plus) (Takara Bio, USA) with the Applied Biosystems 7500 Real-Time PCR System (Applied Biosystems, CA, USA). Program of real-time PCR consisted of: 95 ℃ for 30s, followed by 40 cycles of 94 ℃ for 15 s and 60 ℃ for 30 s , with a final melting curve analysis from 65℃ to 95 ℃ (0.5 ℃ increments). The cycle time values were normalized to GAPDH expression in the same sample as an internal control. The gene expression levels were calculated using the 2 - ΔΔCt method. Each sample was analyzed in triplicate, and the relative expression of mRNA was calculated after normalization to GAPDH. The primer sequences used for amplification were as follows: GAPDH, F: 5′-GGAAGCTTGTCATCAATGGAAATC-3′, R: 5′-TGATGACCCTTTTGGCTCCC-3′; ASC, F: 5′-GGATGCTCTGTACGGGAAGG-3′, R: 5′-GATTCAGGATGATTTGGTGGG-3′; AIM2, F: 5′-AACCCCGAAGATCAACACGC-3′, R: 5′-CATTGTGTCCTCGTTTCTAACCC-3′; GSDMD, F: 5′-CTGGTTATTGACTCTGACTTGGACG-3′, R: 5′-TGTCAGGAAGTTGTGGAGGCA-3′; IL-1β, F: 5′- TACCTGTCCTGCGTGTTGAAA-3′, R: 5′-GGTGCTGATGTACCAGTTGGG-3′; IL-18, F: 5′-TGCACCCCGGACCATATTTAT-3′, R: 5′-TTGCATCTTATTATCATGTCCTGGG-3′; CASP1: F: 5′-TCGCTTTCTGCTCTTCCACA-3′; R: 5′-GGCATCTGCGCTCTACCATCT-3′.

### Primary hippocampal neuron culture

Hippocampal neurons were isolated from E17 SD rat embryos. The foetal brains were immersed in DMEM/F12 medium and the hippocampus was dissected under a microscope. Collected hippocampus tissue was digested with 2 mg/mL papain (Macklin, Shanghai, China) and 0.1 mg/mL DNase (Macklin, Shanghai, China) at 37℃ for 30 min. The tissue was triturated to release cells, and the resulting suspended cells was collected. Neuron fractions were isolated by OptiPrep density gradient (Serumwerk Bernburg AG, Bernberg, Germany) following the manufacturer**'**s instructions. Purified neurons were resuspended and seeded on poly-L-lysine-coated coverslips (0.1 mg/mL) (Sigma, MA, USA) at 7 × 10^5^ cells/cm^2^. Cultures were maintained in a humifified incubator at 37 °C with 5% CO₂. After 1 h, medium was replaced with neurobasal medium containing GlutaMAX and B-27 (all from Gibco, California, USA). Experiments were conducted at 7 days *in vitro*.

### Primary microglia culture and treatment

Microglia were isolated from cerebral cortices of 1-day-old neonatal SD rats. Cortical tissues were digested with 0.25% trypsin-EDTA (Gibco, California, USA) for 20 min at 37 °C, mechanically triturated in DMEM/F12 medium, and filtered through 70-μm mesh cell strainer. Cells were plated in uncoated flasks (Corning, Arizona, USA)) with DMEM/F12 containing 10% FBS (Gibco, California, USA). After 14 days *in vitro*, microglia were separated from mixed glial cultures by orbital shaking (220 rpm, 37 °C, 1 h). Enriched microglia were seeded onto PDL-coated plates for subsequent treatments.

On the next day, recombinant lentivirus with the AIM2-knockout plasmid (Genomeditech, Shanghai, China) was added to the cells (MOI=20). After 24 h of transfection, the cells were cultured with virus-free fresh cell culture medium for another 48 h. To simulate CCH *in vitro*, a chronic hypoxic and hypoglycaemic culture environment was constructed using low-glucose DMEM (1 g/L glucose) (Gibco, California, USA) and a three-gas incubator (Thermo Scientific, Massachusetts, USA), with parameters set to 37 °C, 3% O_2_, 5% CO_2_, 92% N_2_. The optimal time of stimulation and optimal treatment concentration of M2exo@HMPB were determined according to the CCK-8 results. For Poly(dA:dT) (Cat#tlrl-patc, InvivoGen, San Diego, US) induction, the experiment was conducted in DMEM/F12 with final concentration of complexed poly(dA:dT) 1μg/mL medium for 24 h. To compare the effects of probenecid and M2exo@HMPB on AIM2 inflammasome inhibition, microglia were treated with PBS, M2exo@HMPB (80 ppm), or Probenecid (30 ppm)(Sigma-Aldrich, St. Louis, MO, USA). The effects of these treatments were systematically evaluated using immunofluorescence (to assess cellular morphology and marker expression), and ELISA (to quantify inflammatory cytokines).

### Primary microglia and neuron co-culture

To assess M2exo@HMPB-treated microglia effects on neurons, a transwell system (0.4 μm pore inserts, Cat#3470, Corning, Arizona, USA) was used, separating neurons (lower chamber) from microglia (upper chamber). Microglia were pretreated under CCH conditions with or without M2exo@HMPB for 48 h, then co-cultured with neurons for 48 h in a mixed medium (neurobasal medium containing 10% FBS and DMEM/F12 containing B27, 4:1 ratio). Neurons were fixed with 4% PFA and immunostained with an anti-MAP2 antibody (1:300, Cat#MAB3418, Millipore, MA, USA). Supernatants were analyzed for IL-1β and IL-18 levels via ELISA.

### BODIPY 493/503 fluorescence staining

To establish an *in vitro* model of hepatic steatosis, HepG2 cells were treated with 1 mM FFA (OA and PA at a 2:1 vol ratio, all from Macklin, China) in a complete medium containing 1% fatty acid-free BSA for 24 h. Control cells were treated with 1% fatty acid-free BSA.

For Bodipy staining, HepG2 cells treated with 1 mM FFA for 24 h were washed twice with PBS and fixed with 4% paraformaldehyde for 10 min at room temperature. Cells were stained with 1 μM Bodipy 493/503 (Servicebio, Wuhan, China) and then incubated at 37 ℃ for 1 h in the dark. The nucleus was stained with DAPI. Cells were then washed three times with PBS and photographed using a fluorescence microscope (Olympus IX53, Tokyo, Japan).

### Oil Red O staining

The working solution was prepared by mixing Oil Red O stock solution (Sigma-Aldrich, St. Louis, MO, USA) with distilled water (3:2), incubated for 20 min, and filtered (0.22 μm). Treated HepG2 cells were washed twice with PBS, fixed with 4% paraformaldehyde for 10 min and stained with Oil Red O for 1 h. Plates were washed three times with water, dried, and imaged.

### SEM

Following treatments, cells were enzymatically digested and resuspended in electron microscope fixative (Servicebio, Wuhan, China). After fixation at room temperature for 2 h, the cells were dehydrated and finally dried via tert-butanol dehydration. Subsequently, the cells were sputter-coated with gold and imaged under the Hitachi S-4800 scanning electron microscope (Hitachi, Ltd., Tokyo, Japan).

### Cell viability assays

Cell viability was assessed by Cell Counting Kit-8 (Beyotime, Shanghai, China). Following the manufacturer**'**s instructions, primary rat microglia cells were seeded onto 96-well plates and cultured overnight. Cells were then incubated with different concentrations (0, 10, 20, 40, 80 μg/mL) of M2exo@HMPB for another 24h. 10 μL of CCK-8 solution was added to each well and incubated for 1h at 37 °C. The absorbance of each well was measured at 450 nm using a microplate reader (Thermo Fisher Scientific, MA, USA).

### Calcein-AM and propidium iodide (PI) assays

To assess the impact of different treatments on cell survival, cells were stained using a live-dead assay kit (Yeasen Biotech, Shanghai, China). According to the manufacturer**'**s instructions, the live cells were incubated with Calcein-AM and PI solutions for 15 min at 37 °C. Images were acquired using a fluorescence microscope (IX53, Olympus, Tokyo, Japan) by an investigator blinded to experimental groups.

### LDH release assay

LDH release in cell culture supernatants was quantified using an LDH Cytotoxicity Assay Kit (Beyotime Biotechnology, Shanghai, China) following the manufacturer's guidelines. The absorbance at 490 nm was detected by a microplate reader (Thermo Fisher Scientific, USA).

### Western blot analysis

Hippocampus tissues from rats were homogenized in cold RIPA buffer (Beyotime, Shanghai, China) containing a mixture of protease and phosphatase inhibitors (Beyotime, Shanghai, China), followed by centrifugation at 12,000 rpm for 30 min at 4 °C. The protein concentrations were assayed using a BCA kit (Epizyme, Shanghai, Shanghai). Different samples with the same amount of protein were separated by sodium dodecyl sulphate‒polyacrylamide gel electrophoresis (SDS‒PAGE) on 7.5% or 12% gels (Epizyme, Shanghai, China) and transferred to polyvinylidene difluoride membranes (PVDF). The membranes were then blocked with 5% BSA for 1 h at room temperature and incubated overnight at 4 °C with primary antibodies as follows: AIM2 (1:2000, Cat#20590-1-AP, Proteintech, Wuhan, China), GSDMD (1:1000, Cat#GB114198, Servicebio, Wuhan, China), ASC (1:1000, A16672, ABclonal, Wuhan, China), caspase1-p20 (1:1000, Cat#A16792, ABclonal, Wuhan, China), and β-actin (1:20000, Cat#66009-1-Ig, ProteinTech, Wuhan, China). The PVDF membranes were washed in TBST and incubated with relevant secondary antibodies at room temperature for 1 h. Then, immunoblots were detected using the ECL kit (Yeasen, Shanghai, China) and quantified semi-quantitatively with ImageJ software.

### H&E and Nissl staining

Paraffin slices were deparaffinized, rehydrated, and subjected to heat-induced antigen retrieval via microwave treatment and blocked with 3% donkey serum and 0.3% Triton X-100 in PBS. Subsequently, the brain sections were stained with haematoxylin and eosin or 0.5% tolyl violet solution and finally covered with neutral resin.

### Luxol-fast-blue staining

LFB staining was used to assess white matter lesion (WMLs) severity. Dewaxed sections were immersed in LFB solution (Cat#G1030, Servicebio, Wuhan, China) at 37 °C overnight. Excess stain was removed by 95% ethanol and deionized water. Differentiation was performed using 0.05% lithium carbonate (Sinopharm, China) for 20 s and 70% ethanol until nuclear decolorization. The WMLs were evaluated in five brain regions: the optic tract, internal capsule, caudoputamen, corpus callosum (Medial) and corpus callosum (Paramedian). The severity of the WMLs was graded as normal (Grade 0), disarrangement of the nerve fibers (Grade 1), the formation of marked vacuoles (Grade 2), and the disappearance of myelinated fibers (Grade 3). The severity of neuropathology was assessed by three researchers blindly.

### Histological staining

The immunohistochemical staining of anti-Iba-1 antibody was performed to assess the microglial activation. Slices were incubated with Iba-1 (1:200, Cat#ab178846, Abcam, Cambridge, UK) overnight at 4 ℃. After triple washing in PBS, horseradish peroxidase (HRP)-conjugated immunoglobulin G (IgG) secondary anti-rabbit antibody (Cat#GB23303, 1:1000, Servicebio, Wuhan, China) was incubated for 1 h at room temperature. The localization and distribution of immunopositive cells in the brain were visualized under a microscope (IX53, Olympus, Tokyo, Japan).

### Transmission electron microscopy (TEM)

Following transcardial perfusion, the CA1 subregion of the hippocampus was dissected into 1 mm^3^ slices and then fixed with electron microscopy fixative (Servicebio, Wuhan, China) overnight at 4 °C. After being washed in 0.1 M PBS three times, slices were post-fixed in 1% OsO_4_ for 2 h at room temperature. After dehydrating through a graded series of ethanol solutions and acetone, the specimens were embedded in resin and polymerized in a 60℃ oven for 48 h. 70-nm ultrathin sections were cut and stained with uranyl acetate and lead acetate. Slices were analyzed by a transmission electron microscope (H-7800, Hitachi, Japan) to examine cellular and subcellular structures.

### Transcriptome sequencing analysis

RNA-seq was conducted by XuRan Biotechnology (Shanghai, China) and according to the manufacturer's protocol. Total RNA of rat hippocampus was extracted to construct a cDNA library and perform RNA-seq. The expression levels of genes were calculated by quantifying the cDNA fragments per kilobase of transcript per million fragments mapped. Data visualization (plots and heatmaps) was performed using an online platform (https://www.xiantaozi.com).

### Statistical analysis

The statistical analyses were performed with GraphPad Prism (version 9.4.1, La Jolla, CA, USA) and R (version 4.3.2). The differences in means among multiple groups were analyzed using one-way or two-way analysis of variance (ANOVA) followed by Dunnett (all conditions compared with an indicated group) or Tukey (comparisons between all conditions) multiple-comparison tests. Data are expressed as the mean **±** standard error of the mean (SEM). A p-value < 0.05 was considered statistically significance.

### Data Availability Statement

The data that support the findings of this study are available from the corresponding author upon reasonable request.

## Supplementary Material

Supplementary figures.

Supplementary Video 1.

Supplementary Video 2.

## Abbreviations

AIM2: Absent in melanoma 2
VaD: vascular dementia
HMPB: hollow manganese Prussian blue
dsDNA: double-stranded DNA
CCH: Chronic cerebral hypoperfusion
BCCAO: bilateral common carotid artery occlusion
CBF: cerebral blood flow
SDS-PAGE: sodium dodecyl sulfate-polyacrylamide gel electrophoresis
WGA: wheat germ agglutinin
HH: hypoxic-hypoglycemic
SEM: scanning electron microscopy
TEM: transmission electron microscopy
NAFLD: non-alcoholic fatty liver disease
FFA: free fatty acid
BBB: blood-brain barrier
GSEA: Gene Set Enrichment Analysis
