# Supplementary material for: An AIM2 inflammasome biomimetic mineralization inhibitor for vascular dementia therapy
Source: Theranostics. 2025 May 25;15(13):6347–68. doi: 10.7150/thno.110389 (PMC12159838; doi:10.7150/thno.110389)
Supplement: Supplementary file 1 — Supplementary figures. [file thnov15p6347s1.pdf]

Supporting Information

An AIM2 inflammasome biomimetic mineralization inhibitor for vascular dementia therapy

Yueqi Zhang[a,d]‡, Lixian Jiang[b,c]‡, Rongrong Wu[b]‡, Wei Gao[c], Xiaojie Zhang[a,d], Lan Liu[a,d], Yaxuan Zhang[a,d], Jin Lu[e], Yuanyi Zheng[b,c]*, Xiaojun Cai[b,c]*, Jianliang Fu[a,d]**

[a]Department of Neurology, Shanghai Sixth People's Hospital Affiliated to Shanghai Jiao Tong University School of Medicine, Shanghai 200233, P. R. China

[b]Shanghai Key Laboratory of Neuro-Ultrasound for Diagnosis and Treatment, Shanghai 200233, P. R. China

[c]Department of Ultrasound in Medicine, Shanghai Sixth People's Hospital Affiliated to Shanghai Jiao Tong University School of Medicine, Shanghai 200233, P. R. China

[d]Shanghai Neurological Rare Disease Biobank and Precision Diagnostic Technical Service Platform, Shanghai 200233, P. R. China

[e]Department of Pharmacy, Shanghai Sixth People's Hospital Affiliated to Shanghai Jiao Tong University School of Medicine, Shanghai 200233, P. R. China

Corresponding author:

Jianliang Fu: fujianliang@163.com; Xiaojun Cai: c1x2j34@163.com or caixiaojun00@sjtu.edu.cn; Yuanyi Zheng: zhengyuanyi@sjtu.edu.cn ; Wei Gao: 1033452945@qq.com

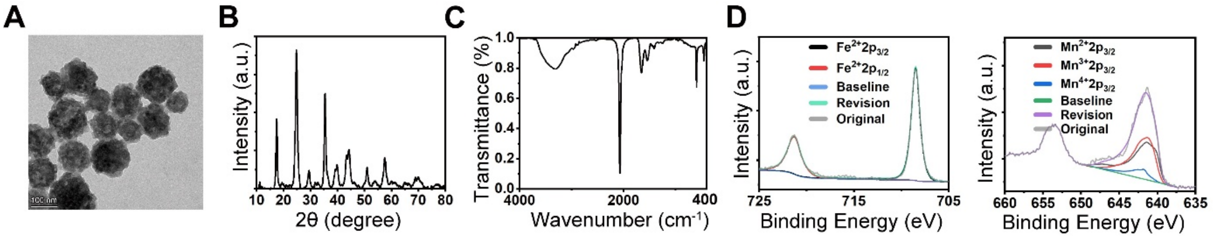

Figure S1. Characterization HMPB. A) Representative Transmission electron microscopy (TEM) images of HMPB. Scale bar: 100nm. **B)** X-ray diffraction spectrum (XRD) of HMPB. **C)** Fourier transform infrared spectroscopy (FTIR) of HMPB. **D**) X-ray photoelectron spectroscopy (XPS) binding energy peaks of Fe and Mn in HMPB.

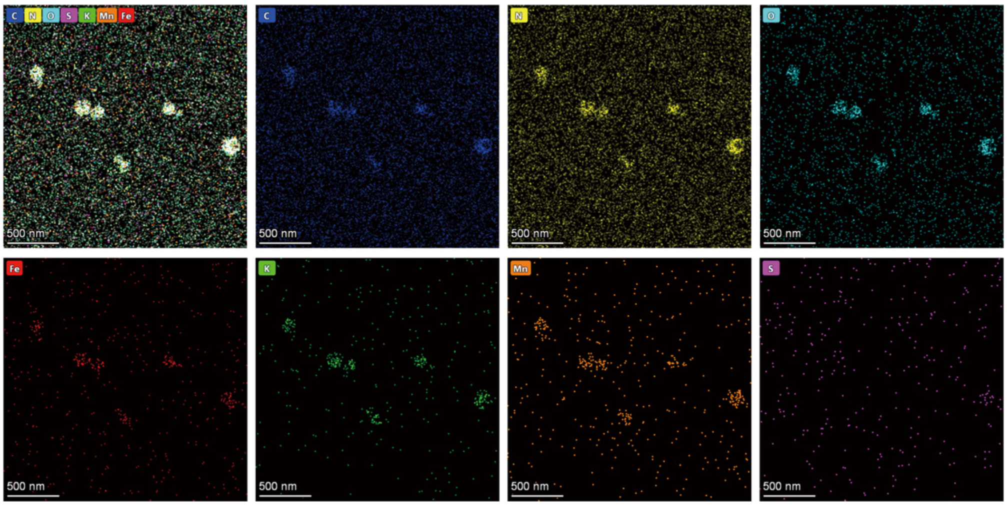

Figure S2. The element mapping of HMPB (C, O, N, K, Fe, Mn, and S). Scale bar: 500 nm.

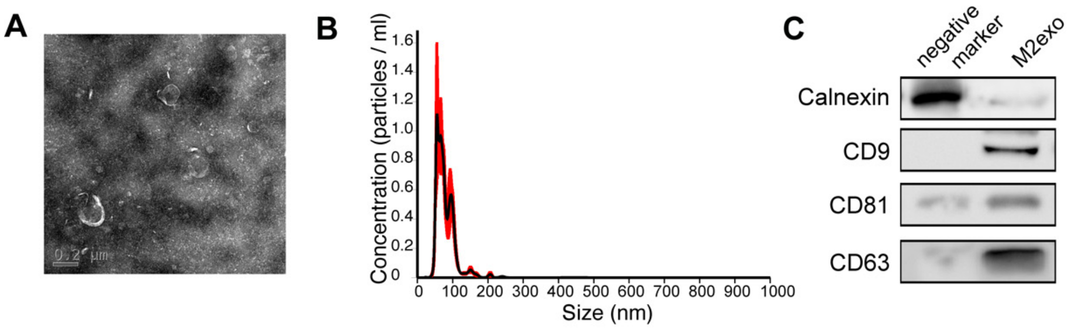

Figure S3. Characteristics of M2 macrophage-derived exosomes (M2 exosomes). A) Representative TEM image of M2 exosome. Scale bar: 0.2 μm. **B**) Nanoparticle tracking analysis (NTA) of M2 exosome. **C**) Western blot analysis of exosomal markers (CD9, CD63, and CD81).

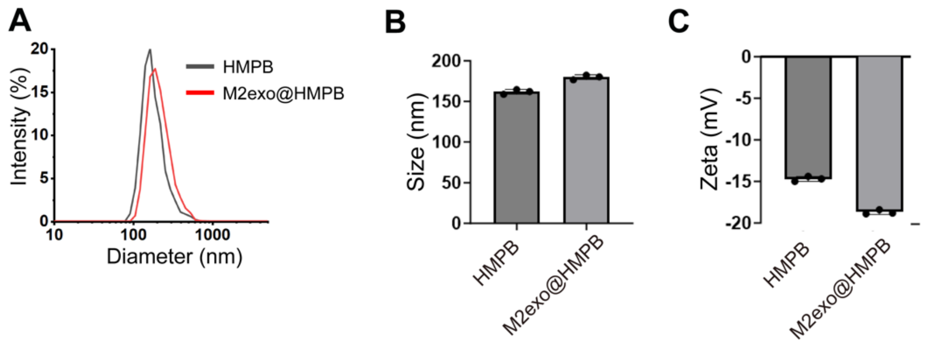

Figure S4. Physicochemical properties of M2exo@HMPB. A) Intensity size distribution profiles of HMPB and M2exo@HMPB by dynamic light scattering (DLS). **B)** Hydrodynamic diameter of HMPB and M2exo@HMPB by DLS. **C)** Zeta potentials of HMPB and M2exo@HMPB.

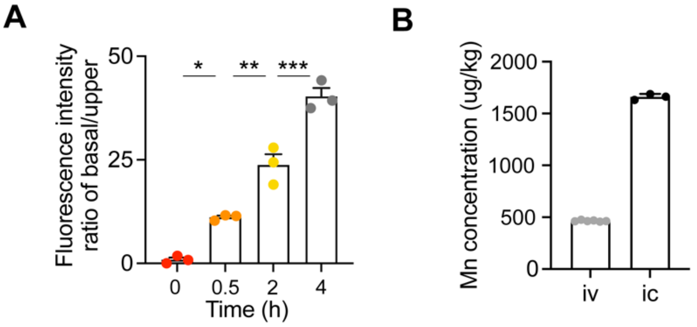

Figure S5. ***In vitro*** **and** ***in vivo*** **biodistribution of M2exo@HMPB. A)** Blood-brain barrier (BBB) translocation efficiency of M2exo@HMPB *in vitro*. n=3 per group. **B)** Comparative brain Mn levels 24 h post intravenous (*iv*) versus intracerebroventricular (*ic*) administration. n = 3-6 per group. Data: mean ± SEM. *P < 0.05; **P < 0.01; ***P < 0.001.

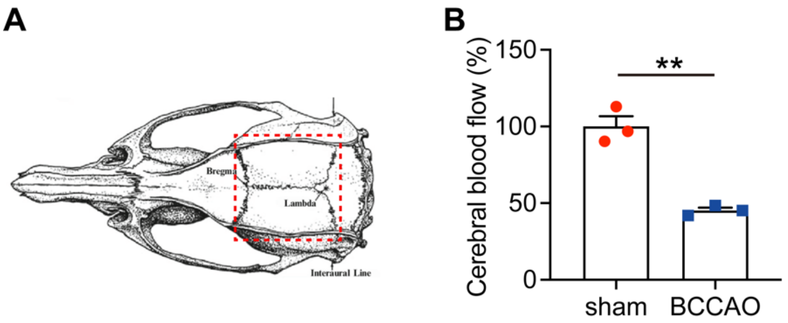

Figure S6. Validation of chronic cerebral hypoperfusion (CCH) rat model via bilateral common carotid artery occlusion (BCCAO). A) Schematic of laser speckle imaging (LSI) detection region. **B)** Quantification of cerebral blood flow (CBF) before and after BCCAO surgery. The CBF decreased significantly to approximately 50% of the baseline. n = 3 per group. Data: mean ± SEM. *P <0.05, **P < 0.01, ***P < 0.001.

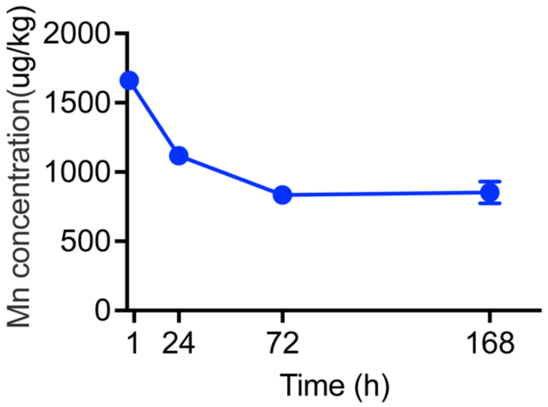

Figure S7. Long-term *in vivo* metabolic profile of M2exo@HMPB. Data: mean ± SEM. n=3-6/group.

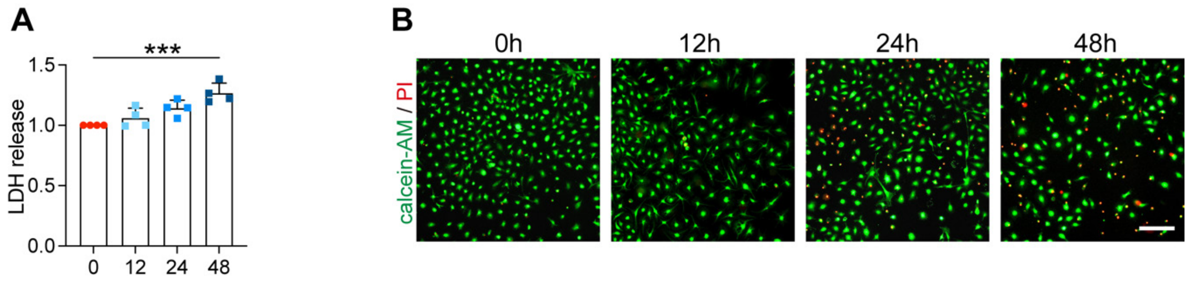

Figure S8. Hypoglycemia/hypoxia (HH)-induced microglial damage. A) Time-dependent LDH release under HH conditions. n = 4 per group. Data: mean ± SEM. *P < 0.05, **P < 0.01, ***P < 0.001. **B)** Live/dead staining of primary microglia (calcein AM [green]/propidium iodide [red]) post-HH treatments. n = 3 per group. Scale bar: 50 µm.

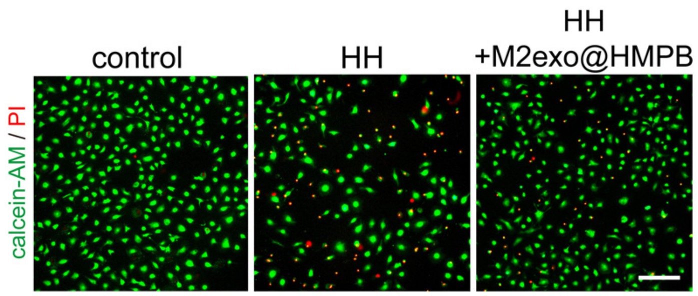

Figure S9. M2exo@HMPB mitigates prolonged HH-induced microglial death. Live/dead staining of primary microglia post-treatments. Scale bar: 50 µm. n = 3 per group.

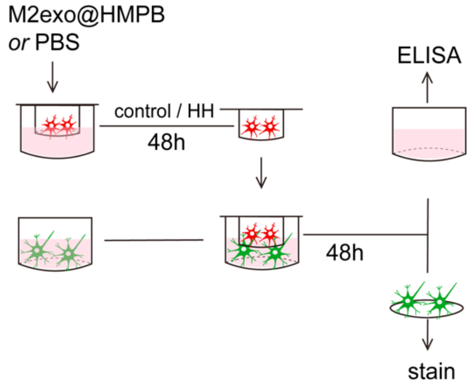

Figure S10. Experimental design for microglia and neuron co-culture.

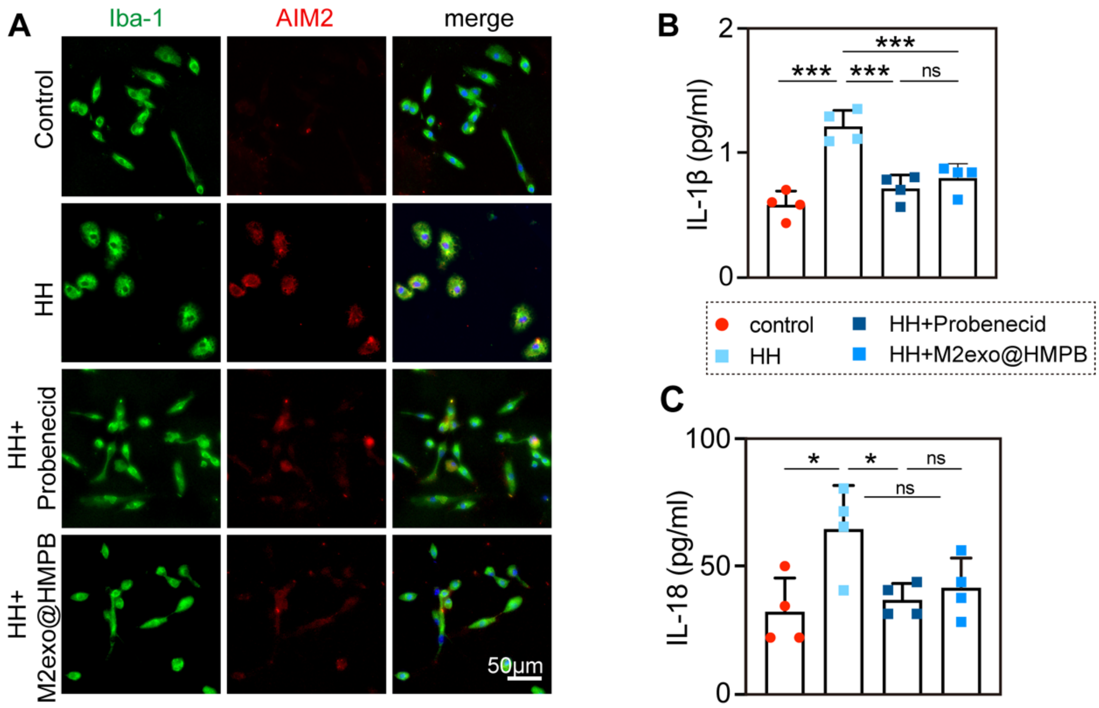

Figure S11. AIM2 inflammasome modulation by M2exo@HMPB. A) AIM2 (red)/Iba-1 (green) co-staining in microglia. Scale bar: 50μm, n = 3 for each group. **B**) IL-1β and **C**) IL-18 levels in supernatant by ELISA. n = 4. Data: mean ± SEM. *P < 0.05; **P < 0.01; ***P < 0.001. ns: not significant.

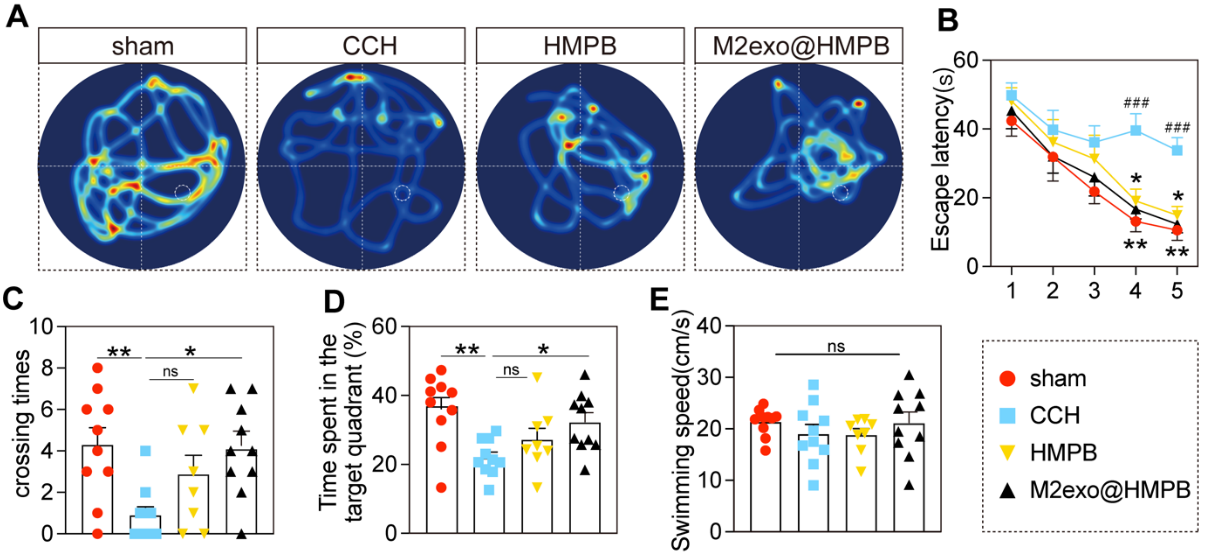

Figure S12. Intracerebroventricular M2exo@HMPB improves cognitive deficits in CCH rats. A) Representative Morris water maze swim paths (probe trial). **B)** Escape latency during hidden platform training. **C)** Platform crossings and **D)** Target quadrant occupancy in probe trial. **E)** Swimming speeds. n = 8-10 per group. Data: mean ± SEM. *P < 0.05, **P < 0.01, ***P < 0.001 vs. CCH; #P < 0.05, ##P < 0.01, ###P < 0.001 vs. sham; ns: not significant.

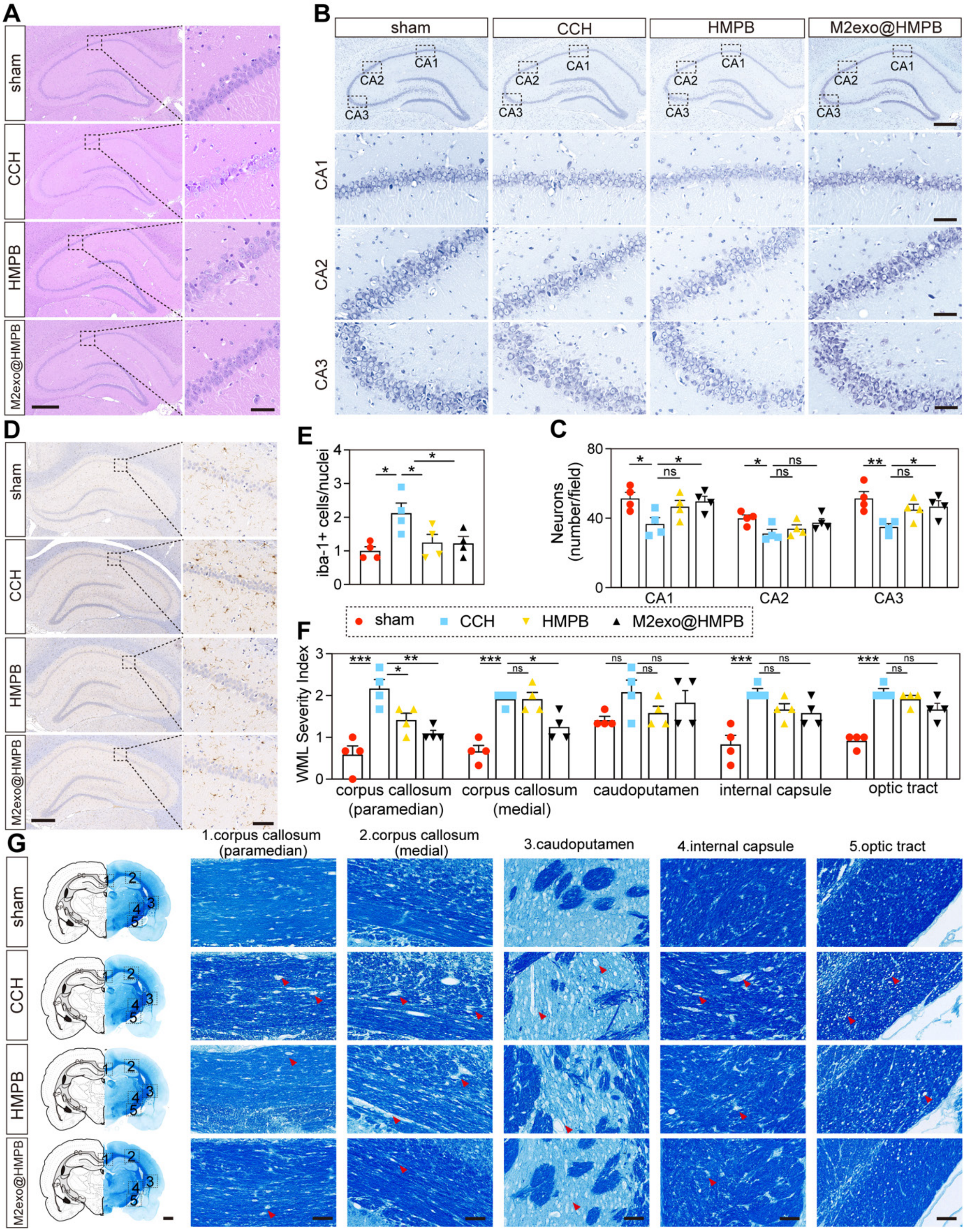

Figure S13. Effect of intracerebroventricular M2exo@HMPB on the levels of glial activation, white-matter integrity, and hippocampal neuronal density in the hippocampus in CCH rats. A) Representative H&E staining of the rat hippocampus. Scale bar: 500μm (left panel) or 50μm (right panel), n = 3 per group. **B-C)** Nissl staining and neuronal quantification in CA1/CA2/CA3 regions. Scale bar: 500μm (upper panel) or 50μm (lower panels), n = 4 per group. **D-E)** Immunostaining of Iba-1 and Iba-1^{+} microglia quantification. Scale bars: 200μm

(left panel) or 20μm (right panel), n = 4 per group. Representative Luxol fast blue staining **F**) and quantification of white-matter integrity **G**) in the corpus callosum (paramedian), corpus callosum (medial), caudoputamen, internal capsule, and optic tract in rats. Scale bars: 1mm (left panel) or 50μm (right panels), n = 4 per group. Data: mean ± SEM. *P < 0.05; **P < 0.01; ***P < 0.001 vs. CCH. ns: not significant.

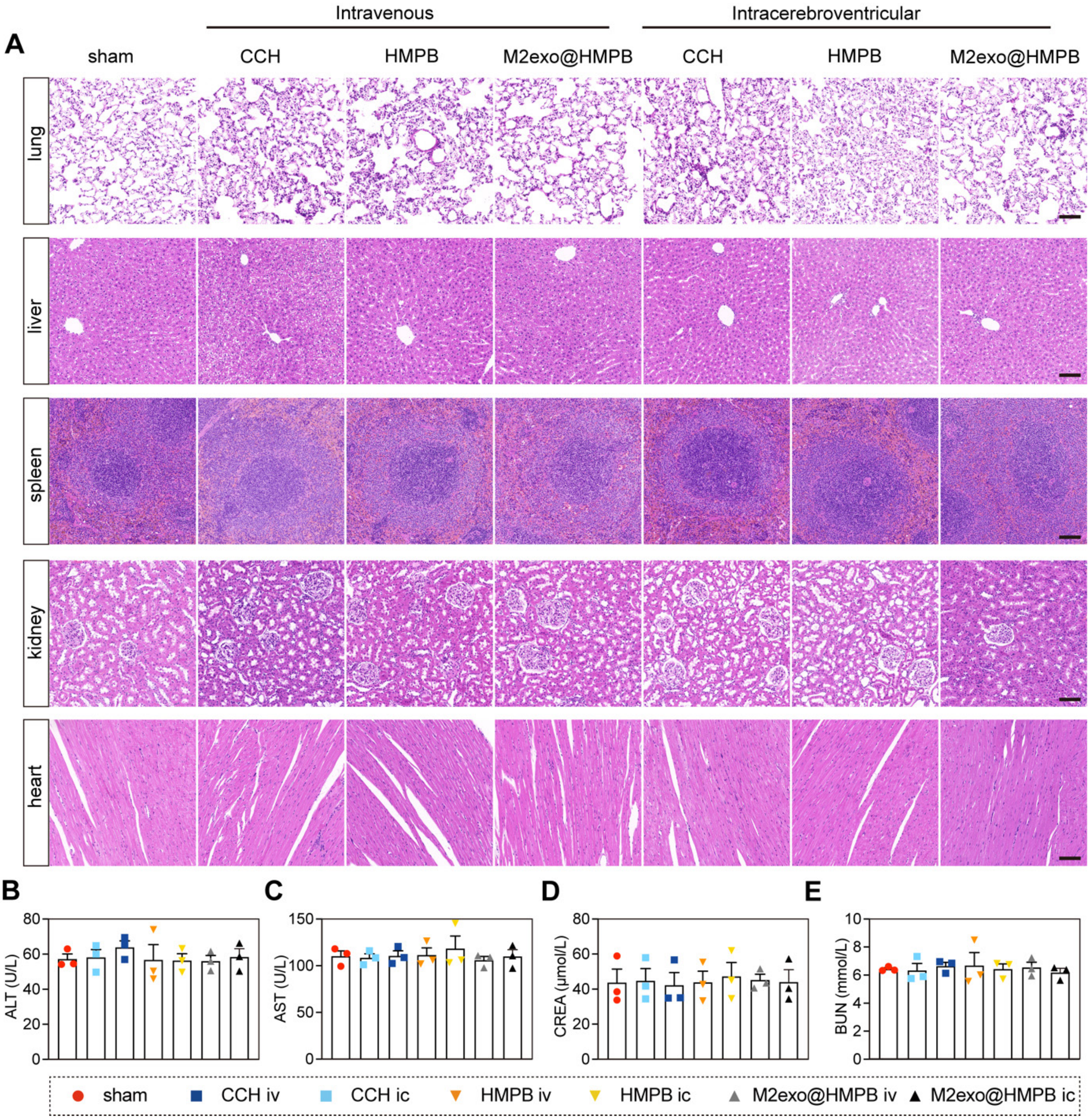

Figure S14. *In vivo* biosafety assessment of nanoparticles. A) H&E-staining major organs. Scale bars = 50 μm. **B)** Serum liver function biochemical markers, alanine aminotransferase (ALT), and **C**) aspartic acid transferase (AST). **D**) Serum kidney function biochemical markers, Creatinine (Cre) and E) blood urea nitrogen (BUN). Data: mean ± SEM, n = 3 per group.

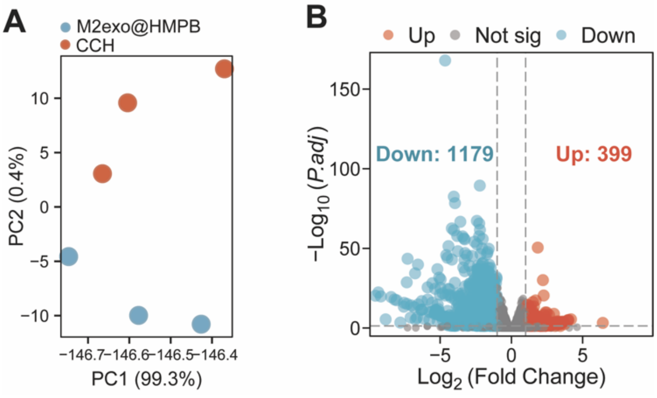

Figure S15. Transcriptomic profiling of CCH vs M2exo@HMPB-treated hippocampi. A) Principal component analysis (PCA) plot of differentially expressed genes (DEGs). **B)** Volcano plot of DEGs (CCH vs M2exo@HMPB).

Supplementary Movies

Movie S1: MD of AIM2^{HIN} domain with manganese ferrocyanide interface.

Movie S2: MD of AIM2^{PYD} domain with manganese ferrocyanide interface.
